# Structural Basis for the Enzymatic Activity of the HACE1 HECT‐Type E3 Ligase Through N‐Terminal Helix Dimerization

**DOI:** 10.1002/advs.202207672

**Published:** 2023-08-03

**Authors:** Sunil Singh, Satoru Machida, Nikhil Kumar Tulsian, Yeu Khai Choong, Joel Ng, Srihari Shankar, Yaochen Liu, Krisha Vashdev Chandiramani, Jian Shi, J Sivaraman

**Affiliations:** ^1^ Department of Biological Sciences National University of Singapore 14 Science Drive 4 Singapore 117558 Singapore; ^2^ Department of Biochemistry National University of Singapore 28 Medical Drive Singapore 117546 Singapore

**Keywords:** cancer, HACE1, HECT E3 ligases, RAC1, ubiquitination

## Abstract

HACE1 is an ankyrin repeat (AKR) containing HECT‐type E3 ubiquitin ligase that interacts with and ubiquitinates multiple substrates. While HACE1 is a well‐known tumor suppressor, its structure and mode of ubiquitination are not understood. The authors present the cryo‐EM structures of human HACE1 along with in vitro functional studies that provide insights into how the enzymatic activity of HACE1 is regulated. HACE1 comprises of an N‐terminal AKR domain, a middle (MID) domain, and a C‐terminal HECT domain. Its unique G‐shaped architecture interacts as a homodimer, with monomers arranged in an antiparallel manner. In this dimeric arrangement, HACE1 ubiquitination activity is hampered, as the N‐terminal helix of one monomer restricts access to the C‐terminal domain of the other. The in vitro ubiquitination assays, hydrogen‐deuterium exchange mass spectrometry (HDX–MS) analysis, mutagenesis, and in silico modeling suggest that the HACE1 MID domain plays a crucial role along with the AKRs in RAC1 substrate recognition.

## Graphical abstract

The regulatory mechanism of HECT‐type E3 ubiquitin ligase HACE1 is described. The cryo‐EM structure of HACE1 reveals the active monomer and autoinhibited dimer. HACE1 homodimerization is mediated by the N‐terminal helix of one subunit inserted onto the HECT domain small N‐lobe of the other subunit. Deletion of the N‐terminal helix enhances the interaction of RAC1 with HACE1 ankyrin repeat (AKR) domain and leads to enhanced ubiquitination of RAC1 in vitro.

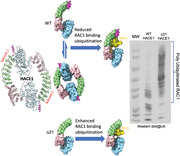

## 1 Introduction

Ubiquitination is a posttranslational modification that affects a variety of cellular events, including protein degradation, cell cycle, transcription, DNA repair, and apoptosis.^[^ ^1^, ^2^, ^3^, ^4^ ^]^ The ubiquitination process involves the sequential enzymatic activities of E1 (ubiquitin‐activating), E2 (ubiquitin‐conjugating), and E3 (ubiquitin ligase) enzymes.^[^ ^5^ ^]^ Ubiquitin is activated by the E1 enzyme, and then complexed with the E2 enzyme through the creation of a E2–Ub thioester. Subsequently, E3‐ubiquitin ligase specifically binds to or interacts with the substrate and the E2–Ub thioester to mediate the ubiquitin transfer to the substrate lysine residue.

E3 ubiquitin ligases are classified into three different families based on the mechanism of ubiquitin transfer: RING‐type (Really Interesting New Gene), RBR‐type (RING‐Between RING), and HECT‐type (Homologous to the E6AP C‐terminus). HECT‐type E3 ligases assemble linkage‐specific polyubiquitin chains on substrate proteins, and their aberrant activity has been linked with multiple diseases, including cancer.^[^ ^6^ ^]^ A total of 28 members make up the HECT E3 ligase family, which is further subdivided into three subfamilies based on their N‐terminal substrate binding domains. There are nine members in the NEDD4 subfamily (containing WW and C2 domains), six in the HERC subfamily (containing RLD domain), and 13 in the “Other” subfamily (containing a variety of N‐terminal domains). These various N‐terminal substrate binding domains are presumably required for interaction with and ubiquitination of a wide range of substrates.^[^ ^4^ ^]^ Sequence identity among HECT E3 ligases varies considerably from 16–92%.^[^ ^7^ ^]^

The catalytic HECT domain (≈40 kDa) is at the C‐terminus, which can be divided into two lobes: the N‐lobe interacts with the E2‐conjugating enzyme, while the C‐lobe contains the catalytic cysteine. Most of the reported crystal structures contain fewer than 100 residues adjacent to the catalytic HECT domain, and little information is available about its conformation in the presence of substrate binding domains.^[^ ^8^, ^9^, ^10^ ^]^ Recently, the structures of both *Nematocida* HUWE1 (287 kDa, 2490 amino acids) and human HUWE1 (482 kDa, 4374 amino acids) were shown to adopt a giant ring architecture. Specifically, the monomeric HUWE1 folds into a ring‐shaped modular architecture with flexibly attached accessory domains.^[^ ^11^, ^12^ ^]^ Yet, with the exception of HUWE1, monomeric HECT E3 ligases have never been reported to form a ring architecture. Besides, the large molecular‐weight gap among HECT‐type E3 ligases makes it difficult to predict the mechanism of their substrate recognition and regulation.

HACE1 (HECT domain and Ankyrin repeat (AKR) containing E3 Ubiquitin Protein Ligase 1) is an AKR‐containing HECT E3 ligase in the “Other” subfamily. HACE1 binds to numerous dissimilar targets with a lack of structural homology, including Optineurin (OPTN), Cyclin C, RAC1, Rab11, β2AR adrenergic receptor, and Spindlin1. HACE1 is a tumor suppressor E3 ligase that has been reported to inhibit breast cancer cell proliferation through 26S proteasome‐mediated degradation of RAC1.^[^ ^13^, ^14^, ^15^, ^16^, ^17^, ^18^, ^19^ ^]^ It also ubiquitinates autophagy receptor OPTN to initiate autophagy in lung cancer.^[^ ^15^ ^]^ HACE1 expression levels vary between sporadic Wilms’ kidney cancer and normal kidney cancer, and its activity has been shown to be essential for the optimal upregulation of NRF2 under oxidative stress.^[^ ^20^ ^]^ HACE1‐mediated NRF2 upregulation of oxidative stress in brain tissues maintains redox homeostasis, which may suggest a role for HACE1 in NRF2‐mediated antioxidative stress response pathways in neurodegenerative diseases.^[^ ^21^ ^]^ Additionally, HACE1 inactivation impairs TNFR1‐mediated NF‐kB activation and apoptosis, which leads to necroptosis.^[^ ^22^ ^]^

Sequence homology analysis across kingdoms shows human HACE1 (909 amino acids) as being closest in homology to human HUWE1 (4375 amino acids).^[^ ^23^ ^]^ Yet, despite their phylogenetic closeness, HACE1 and HUWE1 share homology only in the HECT domain (≈42% sequence identity). In cellular and clinical studies, HACE1 pathways are shown to have therapeutic potential; however, as yet, there has been no clear mechanism to explain HACE1 substrate recognition and regulation.^[^ ^24^, ^25^ ^]^ Therefore, a structural and biochemical approach is needed to understand HACE1 mechanism of action.

Here, we report the cryo‐EM structures of monomeric and dimeric HACE1 along with in vitro biochemical assays. We show that HACE1 adopts a unique G‐shaped architecture and comprises an N‐terminal AKR domain, a middle (MID) domain, and a C‐terminal domain (HECT). HACE1 forms a homodimer in an antiparallel orientation through its N‐terminal helix (aa 1–21), and this prevents access to the C‐terminal HECT domain of the second monomer in the dimer. Deleting the N‐terminal helix breaks the HACE1 dimer into monomers. Remarkably, monomeric HACE1 displays enhanced ubiquitination activity compared with dimeric HACE1. Further, we show a role for the C‐terminal residues in HACE1‐mediated auto‐ and substrate ubiquitination. Using hydrogen‐deuterium exchange mass spectrometry (HDX–MS), we mapped the interaction interface between HACE1 and substrate RAC1. In sum, we show that HACE1 regulates its activity by dimerization, with RAC1 substrate recognition mediated by its MID domain and AKRs. The results of our study provide mechanistic insight into how “Other” subfamily HECT type E3 ligases may control their enzymatic activity.

## 2 Results

### 2.1 Single‐Particle Reconstruction of HACE1

**Figure 1:**
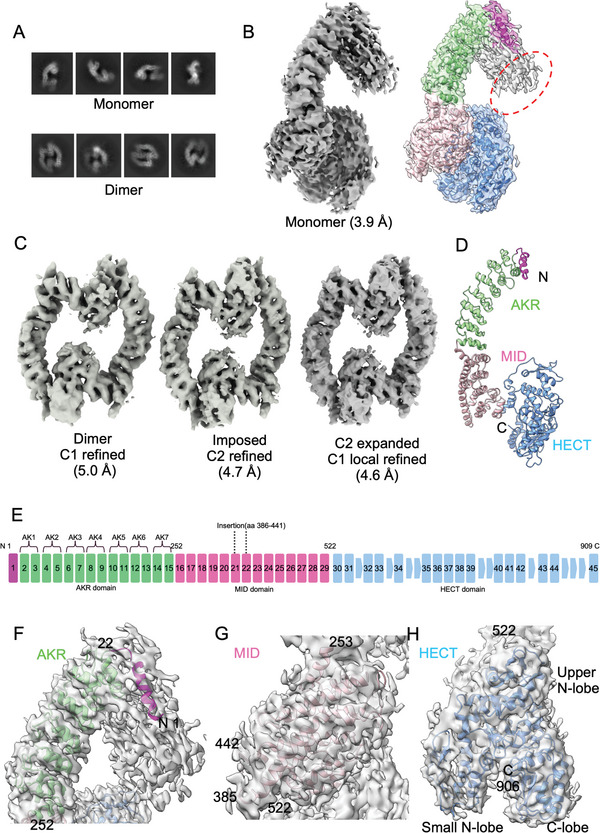
The cryo‐EM structure of HACE1. A) A representative 2D class averages of HACE1 monomer and dimer. B) Coulomb potential map of HACE1 monomer. Red dotted circle indicates the additional density that could not be modeled. C) Coulomb potential map of the HACE1 dimer. A 4.6 Å map was used for modeling. D) Overall structure of HACE1 monomer, including the N‐terminal helix (magenta), the ankyrin repeat (AKR) repeat domain (light green), the middle (MID) domain (pink), and the HECT domain (sky blue). E) Domain organization of HACE1 monomer. The square and the number inside represent helices counted from the N‐terminus. F) The N‐terminal helix and AKR domain model (aa 1–252) fit in monomer map. G) The MID domain model (aa 253–522) fit in monomer map. The insertion loop (aa 386–441) could not be modeled. H) The HECT domain (aa 523–909) fit in monomer map. The last C‐terminal three residues (aa 907–909) could not be modeled.

We determined the cryo‐EM structure of full‐length human HACE1 as a dimer and as a monomer. The cryo‐EM datasets were collected by FEI Titan Krios using purified full‐length HACE1. Full‐length HACE1 eluted as dimer in size‐exclusion chromatography. However, micrographs displayed a mixture of monomers and dimers. Through 2D classification, we identified a G‐shaped HACE1 monomer and a circular dimer in which the subunits faced each other in an anti‐parallel orientation (**Figure** 1A). Coulomb potential map of the monomer was slightly larger than the predicted model, indicating an additional density attached to the N‐terminus (red dotted circle, Figure 1B). Using MS analysis, we confirmed that this density was caused by the uncleaved GST tag. Global resolution was 3.9 Å for the monomer and 4.6 Å for the dimer by gold standard FSC using a 0.143 cutoff (Figure 1B,C and Figures S1, S2, Supporting Information, **Table** **1**).

**Table 1:** Structure determination statistics of HACE1 monomer and dimer

Data collection	OpticsGroup1	OpticsGroup2
EM equipment	Krios	Krios
Voltage [kV]	300	300
Detector	K3	K3
Pixel size [Å]	0.8584	0.8584
Exposure time [s]	60	90
Dose per frame [e^−^ Å^−2^]	1.2	1.8
Defocus range [µm]	0.5 to 2.5	0.5 to 2.5
Number of micrographs	10 296	4529
Number of frames	50	50
Reconstruction	Monomer	Dimer
Software	Relion‐3.1.3	CryoSPARC‐3.2.0
Number of particles used	269 682	71 331
Overall resolution [Å], FSC 0.143 threshold	3.92	4.55
Symmetry	C1	C2
Model building and composition		
Software	Flex‐EM(ccpem‐1.6.0)	Flex‐EM(ccpem‐1.6.0)
	ISOLDE‐1.0.1	ISOLDE‐1.0.1
Modeled residues	850 of 909	1690 of 1818
Refinement Software	Phenix‐1.13	Phenix‐1.13
Mask_CC	0.762	0.772
Volume_CC	0.748	0.765
Atom mask radius [Å]	3.98	4.60
Validation		
MolProbity overall score	1.65	1.72
Clash score, all atoms	3.65	4.49
Rotamer outliers [%]	0	0.27
Ramachandran‐plot statistics [%]		
Favored [% overall]	91.84	91.68
Outlier [% overall]	0	0
R.m.s. deviations		
Bond length [Å]	0.011	0.009
Bond angle [°]	0.842	1.017

### 2.2 HACE1 Structure

HACE1 is composed of an AKR domain at its N‐terminus (aa 26–252), a MID domain (aa 253–522) in the middle of the protein, and a HECT domain at its C‐terminus (aa 523–909) (Figure 1D,E). We observed that a 56‐residue loop (MID‐loop, aa 386–441) in the MID domain is disordered and could not be modeled (Figure 1E,G). The AKR domain that is located at the N‐terminal consists of seven repeats of tandem helices that are predicted to mediate protein‐protein interactions (Figure 1F).^[^ ^26^ ^]^ The MID domain consists of diagonally stacked helices of uneven lengths (Figure 1G). Previous structural analysis has shown that the HECT domain consists of an N‐lobe (upper N‐lobe aa 522–675 and small N‐lobe aa 676–751) and a C‐lobe (aa 752–909).^[^ ^4^ ^]^ The HACE1 HECT domain forms an inverted Y‐shape conformation wherein the C‐lobe is situated close to the N‐lobe small subdomain, the putative binding site for the E2‐conjugating enzyme (Figure 1H). InterProscan software predicted the presence of the N‐terminal AKR domain and the C‐terminal HECT domain in HACE1 based on the sequence homology but predicted no specific domain in between these two primary domains.^[^ ^27^ ^]^

Next, a DALI search found no structural homologs for the full‐length HACE1. However, a search with independent domains found specific homologs for the AKR and HECT domains as expected (Tables S1, S2 and Figure S3A,B, Supporting Information), but no homolog for the MID domain (Table S3, Supporting Information).^[^ ^28^ ^]^ Although the Armadillo‐like (ARL) domain of HUWE1 is also made of uneven helical repeats, they could not be overlapped with the HACE1 MID domain (Figure S3C, Supporting Information). Therefore, these collective results suggest that the HACE1 MID domain comprises a topology that has not yet been described.

### 2.3 Alpha‐Solenoid Folds of the N‐Terminal Domain

Both the HACE1 AKR domain and the HUWE1 ARL domain adopt the alpha‐solenoid fold at their N‐termini. Notably, the HUWE1 full‐length structure is not superimposable—we visually compared the architectures of HACE1 alongside HUWE1 (Figure S3D, Supporting Information). The full‐length structure of HUWE1 shows that its N‐terminal solenoid armadillo‐like (ARL) domain curls back to make contact with the C‐terminal catalytic HECT domain within the monomer. Comparatively, the HACE1 dimer forms a head‐to‐tail contact between the N‐terminal AKR repeat of one monomer and the C‐terminal catalytic HECT domain of the other. Although the dimeric HACE1 and monomeric HUWE1 rings show similar measurement of 100 × 95 Å dimensions in one‐dimension, the HUWE1 ARL domain is twice as thick as the HACE1 AKR domain (37 Å versus 18 Å) (Figure S3D, Supporting Information). This difference may explain how the dimeric HACE1 ring and monomeric HUWE1 ring have similar diameters despite their large molecular weight differences (106 versus 482 kDa). Moreover, the helices of the HUWE1 ARL domain are heterogeneous in size and contain 4–6 helical turns per helix (Figure S3E, Supporting Information). In contrast, the helices of HACE1 AKR domain display tandem repeats of a constant 2.5‐helical turns. These tandem helices contain histidine residues that face toward the connective loop, which is rich in acidic residues (Figure S3F, Supporting Information). These residues make extensive ionic and dipolar interactions and likely contribute to the rigidity of the solenoid architecture of the AKR domain. This pattern was not observed in the HUWE1 ARL domain solenoid architecture. These features show that the alpha‐solenoid folds of the HACE1 AKR domain are structurally distinct to those of the HUWE1 ARL domain.

### 2.4 Role of N‐Terminal Helix in HACE1 Dimeric‐Monomeric Switching

**Figure 2:**
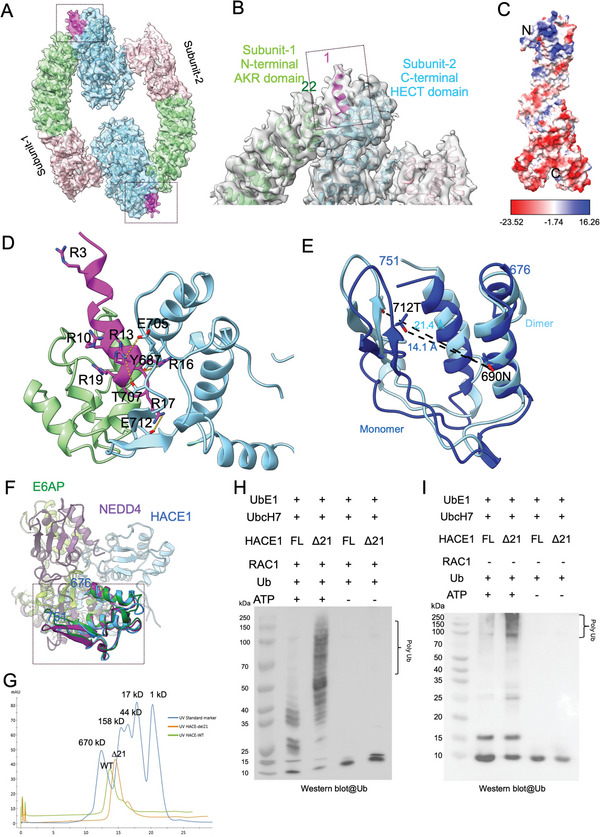
Dimerization of HACE1. A) HACE1 dimer made of two head‐to‐tail contacts between identical subunits. The boxed area is zoomed in on (B). B) The N‐terminal helix plus linker docked with the HECT domain small N‐lobe. The boxed area is zoomed in on (D). C) Electrostatic surface potential map of HACE1 monomer. D) The arginine‐rich N‐terminal helix plus loop (magenta, aa 1–21) is inserted onto the HECT domain small N‐lobe (sky blue) at dimerization interface. The first 19 residues contain six arginine residues as shown. E) Expansion of the HECT domain N‐lobe upon dimerization. Monomer is shown in dark blue. Dimer is shown in sky blue. F) Structural alignment of the HECT domain small N‐lobe from HACE1 (blue), NEDD4 (purple) and E6AP (green). Small N‐lobe is boxed. The small N‐lobes of HACE1 and NEDD4 matched with RMSD 1.035 Å. The small N‐lobes of HACE1 and E6AP matched with RMSD 1.043 Å. G) Analytical gel‐filtration chromatogram of WT HACE1 (aa 1–909) and N‐terminal 21 aa deletion mutant (∆21). H) Western blot analysis of in vitro RAC1 ubiquitination assay using anti‐ubiquitin antibody. WT HACE1 (aa 1–909) and the N‐terminal 21 aa deletion mutant (∆21, aa 22–909) were added at equal concentration. I) Western blot analysis of in vitro autoubiquitination assay with WT HACE1 and ∆21 HACE mutant using anti‐ubiquitin antibody.

Our cryo‐EM structure shows that the HACE1 homodimer makes two head‐to‐tail contacts between the N‐terminal AKR domain of one monomer and the C‐terminal HECT domain of the other (**Figure** **2**A). The N‐terminal helix and the connecting loop dock into the groove of the small N‐lobe of the HECT domain (Figure 2B). The electrostatic surface potential map of the HACE1 monomer shows that the N‐terminal region is highly basic whereas the C‐terminal region is acidic (Figure 2C). In the dimer, residues Arg13, Arg16, and Arg17 of one monomer interacts with Glu705, Tyr687, Glu712, and Thr707 of the other monomer (Figure 2D). There are two interfaces present in the dimer which constituted 2421 and 2418 Å^2^ buried surface areas, respectively, (Table S4, Supporting Information). Consistently, the HECT domain small N‐lobe of the dimer is slightly expanded compared with that of the monomer (Figure 2E, Figure S3G, Supporting Information). A previous report indicated that the small N‐lobes of the HECT domain of two E3 ligases, NEDD4 (PDB: 3JW0) and E6AP (PDB: 1C4Z), were bound by E2‐conjugating enzymes. Structural alignment shows that HACE1 has this equivalent binding site (Figure 2F).^[^ ^29^, ^30^, ^31^ ^]^ In our HACE1 dimer, the N‐terminal helix and connective loop occupy the putative E2‐conjugating enzyme binding site of the other subunit (Figure 2B,D). We hypothesize that obstruction of the small N‐lobe of the HECT domain could hinder the binding and activity of the E2‐conjugating enzyme, thereby modulating HACE1 enzymatic activity. To test this hypothesis, we deleted the N‐terminal 21 residues (∆21) of HACE1 and compared in vitro ubiquitination of RAC1 against the wild‐type (WT) HACE1. Size‐exclusion chromatography and static light scattering indicated that the 21‐residue deletion breaks the dimer into monomers (Table S5, Supporting Information and Figure 2G). Nonreducing SDS–PAGE showed that WT HACE1 has a predominant band at ≈230 kDa which is absent in ∆21 HACE1 lanes (Figure S4A, Supporting Information). Notably the sedimentation velocity analysis of the WT HACE1 using analytical ultracentrifugation showed that 79% of the molecules form dimer or higher oligomers in solution, although higher oligomers were not visible in our cryo‐EM dataset (Figure S4B, Supporting Information). Subsequently, using RAC1 as a target substrate, we performed an in vitro ubiquitination assay, and found that deletion of these N‐terminal residues significantly increased RAC1 ubiquitination (Figure 2H). We also noticed that full length HACE1 showed much weaker autoubiquitination as compared with ∆21 HACE1 (Figure 2I). Together, these findings show that the N‐terminal 21 residues mediate autoinhibitory dimerization, and that disruption of dimerization relieves the restriction on HACE1 activity.

### 2.5 RAC1 Binding Sites

**Figure 3:**
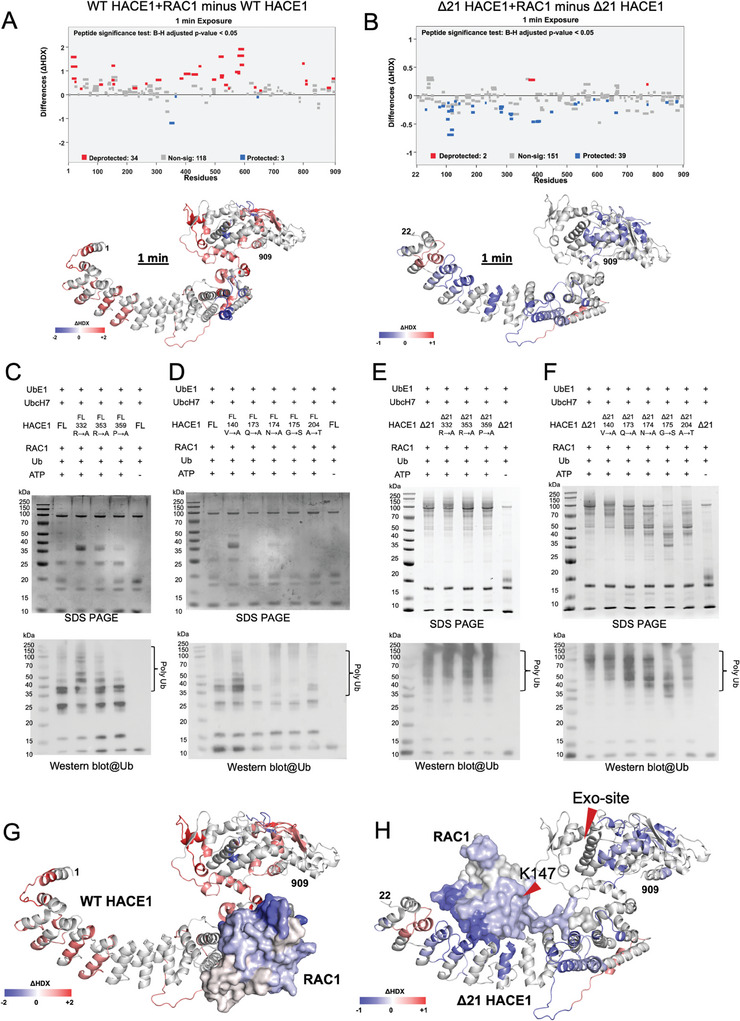
Mapping of HACE1–RAC1 interaction sites. A) Woods’ differential plot showing hydrogen‐deuterium exchange (HDX) differences between WT HACE1 + RAC1 and WT HACE1 alone at 1 min. Peptides (represented as horizontal bars) showing deprotection are in red, while those showing decreased deuterium exchange in WT HACE1 + RAC1 complex are in blue, based on a 99% confidence interval. Differences in deuterium exchange (ΔHDX) were then mapped onto WT HACE1 structure solved in this study and colored as indicated in key (bottom panel). B) Woods’ differential plot showing HDX differences between ∆21 HACE1 + RAC1 and ∆21 HACE1 alone at 1 min. Peptides showing predominantly decreased deuterium exchange (protected, blue lines) were mapped onto structure of ∆21 HACE1 (bottom panel). C) SDS–PAGE and western blot analysis of in vitro RAC1 ubiquitination by WT HACE1 and its middle (MID) domain mutants detected using an anti‐ubiquitin antibody. D) SDS–PAGE and western blot analysis of RAC1 in vitro ubiquitination by WT HACE1 and its ankyrin repeat (AKR) domain mutants detected using an anti‐ubiquitin antibody. E) SDS–PAGE and western blot analysis of in vitro RAC1 ubiquitination by ∆21 HACE1 and its MID domain mutants detected using an anti‐ubiquitin antibody. F) SDS–PAGE and Western blot analysis of RAC1 in vitro ubiquitination by ∆21 HACE1 and its AKR domain mutants detected using an anti‐ubiquitin antibody. G) HDX difference heatmap on in silico model of the WT HACE1 + RAC1 complex. H) HDX difference heatmap on in silico model of the ∆21 HACE1 + RAC1 complex.

Given that disrupted dimerization significantly enhanced HACE1 activity, we surmised that misregulation of HACE1 activity by cancer‐associated mutations may lead to abnormal substrate binding and ubiquitination activity.^[^ ^8^, ^15^, ^32^ ^]^ To this end, we first examined the binding of the target substrate RAC1 to HACE1 and used amide HDX–MS to map the RAC1 interaction sites in the HACE1–RAC1 complex. HDX–MS analysis with WT HACE1 + RAC1 showed decreased deuterium exchange relative to WT HACE1 alone across the peptide cluster in the HACE1 MID domain (aa 327–370), indicating that RAC1 predominantly interacts with the MID domain of WT HACE1 (**Figure** **3**A and Figure S4C, Supporting Information). Our functional studies showed greater ubiquitination of RAC1 by the ∆21 HACE1 construct. Therefore, we also mapped the interaction interface of the ∆21 HACE1 construct bound to RAC1. Notably, HDX–MS analysis with ∆21 HACE1 + RAC1 showed decreased deuterium exchange relative to ∆21 HACE1 alone across the peptide clusters, including AKRs‐2 (peptides covering aa 74–93), repeats‐3 (aa 130–151) and repeats‐6 (aa 231–252), and the N‐terminus of the MID domain (aa 270–311), indicating that RAC1 interacts with both AKR and MID domains of ∆21 HACE1 (Figure 3B). Decreased deuterium exchange was observed in the AKR and MID domains even at the 100‐min labeling timepoint, confirming that ∆21 HACE1 stably associates with RAC1 (Figure S4D, Supporting Information). The intriguing patterns of deuterium exchange by WT‐ and ∆21 HACE1 demonstrate that RAC1 primarily binds to the HACE1 MID domain, and that loss of the N‐terminal helix makes the AKR domain more accessible to RAC1.

Based on these observations, we introduced point mutations at the AKR and MID domains of WT HACE1 and carried out in vitro RAC1 ubiquitination assays. Notably, the Catalogues of Somatic Mutations in Cancer (COSMIC v96, Sanger Institute) lists Arg332 at the WT HACE1–RAC1 interface as the most reported mis‐sense substitution site linked to cancer in HACE1 (Table S6, Supporting Information).^[^ ^33^ ^]^ Our in vitro ubiquitination assay showed that mutation at Arg332 in the MID domain increased RAC1 ubiquitination (Figure 3C). Other mutations in the AKR domain had variable impacts on RAC1 ubiquitination, with decreased ubiquitination activity observed for all mutations except for Val140Ala in the AKR domain, which enhanced RAC1 ubiquitination activity (Figure 3D). Likewise, we made point mutations in ∆21 HACE1 and carried out RAC1 substrate ubiquitination. ∆21 HACE1 showed enhanced activity as compared to WT HACE1. Point‐mutations at the MID domain of ∆21 HACE1 did not impact RAC1 ubiquitination (Figure 3E), whereas point‐mutations at the AKR domain of ∆21 HACE1 affected RAC1 ubiquitination (Figure 3F). Consistent with our HDX–MS analysis, the in vitro ubiquitination assay showed that the role of AKR domain is prominent in ∆21 HACE1. Collectively these results indicate that residues at the HACE1–RAC1 interface have an impact on ubiquitination activity. They also support the notion that HACE1 activity is tightly regulated and that the loss or overexpression of HACE1 alike can lead to protumoral effects in different cancer cells.^[^ ^25^, ^34^, ^35^ ^]^

### 2.6 In Silico Model of HACE1–RAC1 Complex

We next modeled the HACE1 interactions on RAC1 (PDB: 4GZL) using HDX–MS analysis. RAC1 + WT HACE1 showed decreased deuterium exchange relative to RAC1 alone across the peptide clusters covering the residues 21–37, 63–79, and 119–142 (Figure S4E, Supporting Information). Using these interaction interfaces for HACE1 and RAC1, we then performed in silico modeling of the HACE1–RAC1 complex using the ClusPro and Galaxy Web servers.^[^ ^36^, ^37^ ^]^ In two of the predicted models, the HACE1–RAC1 interface corresponded to the peptide fragments that resulted in the highest decrease in deuterium exchange on HACE1 and RAC1 as observed in our HDX–MS analysis. In the model of the WT HACE1 + RAC1 complex, RAC1 associates with the HACE1 MID domain but does not contact the AKR domain (Figure 3G). Notably, our experimental structure of HACE1 MID domain slightly deviates from AlphaFold prediction in the relative angle of stacked helices and the orientation of the connective loop (Figure S4F, Supporting Information). In our experimental model, the loop connecting helices H20 and H21 curls inwardly, leaving a room for RAC1 to make a direct contact with the MID domain without a clash (Figure S4G, Supporting Information). In the model of the ∆21 HACE1 + RAC1 complex, RAC1 contacts both the AKR and MID domains, positioning Lys147 proximal to the upper N‐lobe of the HACE1 HECT domain (Figure 3H). Lys147 is a major ubiquitination site on RAC1.^[^ ^38^ ^]^ This arrangement is analogous to the ubiquitin transfer mechanism of the NEDD4 HECT domain, which is mediated by the sequential transfer of ubiquitin from the small N‐lobe to the upper N‐lobe via the flexible C‐lobe (Figure S4H, Supporting Information) (PDB: 4BBN).^[^ ^30^ ^]^ Using these models, we propose that HACE1 dimer weakly interacts with RAC1 at the MID domain, whereas the monomer (as seen in ∆21 HACE1) has the larger contact area to include the AKR domain to stabilize the HACE1–RAC1 complex.

### 2.7 Molecular Dynamics Simulation of Apo HACE1

As the disruption of dimerization enhanced the interaction at the AKR domain, we sought to understand whether dimerization itself is sufficient to restrict RAC1 access to the AKR domain. AKR‐containing proteins are generally flexible. HUWE1 modulates its activity by self‐closing ring architecture (Figure S3D, Supporting Information).^[^ ^11^, ^12^ ^]^ To verify if monomeric self‐closure could be the case with HACE1, we performed stochastic dynamics simulation of apo HACE1 in coarse‐grained Monte Calo method using CABSflex‐2.0.^[^ ^39^ ^]^ The fluctuation plot shows that the AKR domains of WT and ∆21 HACE1 monomers are equally rigid (Figure S5A,B, Supporting Information), and that N‐terminal helix does not interact with the other domains within HACE1 monomer. To scrutinize the simulated model of WT HACE1 monomer, we performed all‐atom molecular dynamics simulation in 100 ns OPLS force field using Gromacs‐2023.1.^[^ ^40^, ^41^ ^]^ The all‐atom dynamics simulation affirmed the outcome of coarse‐grained Monte Calo method in physiologically relevant timescale (Figure S5B, Supporting Information). We then used CABSflex‐2.0 on WT HACE1 dimer to cross‐check whether HACE1 dimer is flexible enough to allow its AKR domain access to RAC1. Notably, we found that the ring shaped HACE1 dimer does not expand but rather it shrinks inwardly, which was consistent with 100 ns molecular dynamic simulation in OPLS force field (Figure S5C, Supporting Information). Our cryo‐EM structure shows that HACE1 dimer has only 17 Å gap between the N‐terminal AKR domain of one subunit and the C‐terminal HECT domain of other subunit, whereas the dimensions of RAC1 spans 30–40 Å (Figure S5D, Supporting Information), indicating a severe inevitable clash. These findings indicate that HACE1 dimer is very unlikely to interact with RAC1 at the AKR domain. Further, the N‐terminal helix is not involved in intramolecular contacts, so that the self‐closed conformation of WT HACE1 monomer is physically unrealistic. Besides, we tested whether RAC1 causes HACE1 dimer to dissociate into monomer, by nonreducing SDS–PAGE with and without RAC1. The mobility of HACE1 corresponded to the size of dimer despite presence of RAC1 (Figure S5E, Supporting Information). This observation affirms that HACE1 dimerization hinders the RAC1 binding but RAC1 does not affect HACE1 dimerization.

### 2.8 C‐Terminal Residues are Critical for HACE1 Ubiquitination Activity

**Figure 4:**
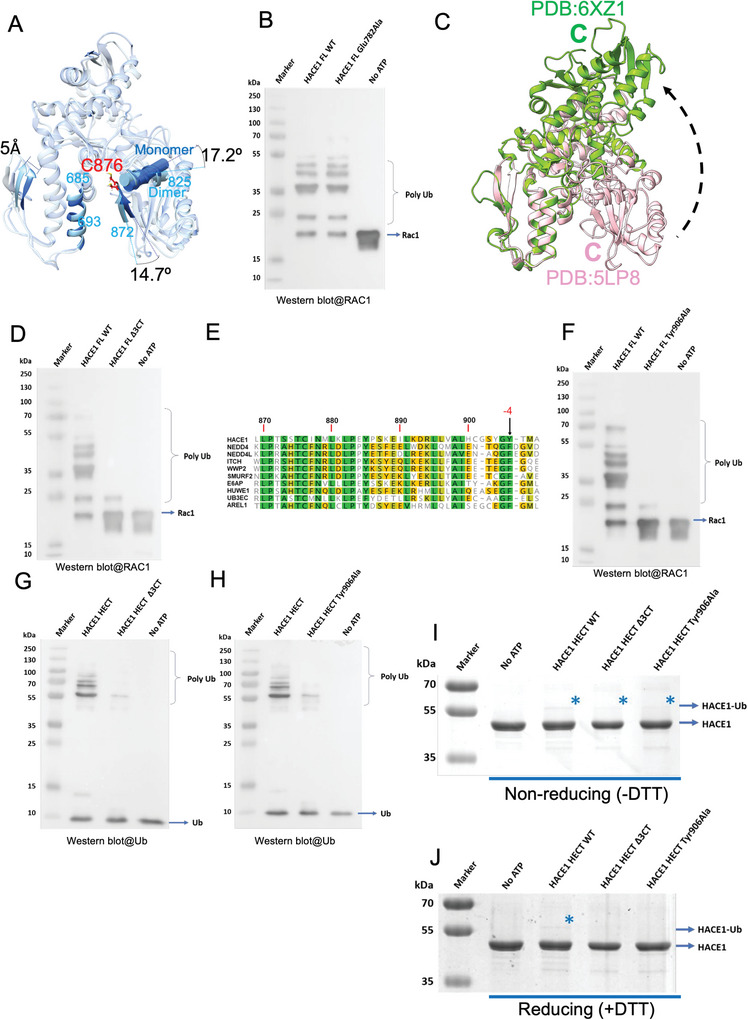
The role of the C‐terminal tail on ubiquitination activity. A) Pair‐wise structural alignment of the HECT domains of HACE1 monomer and dimer. The catalytic cysteine (C876) is highlighted with red. The experimental models are matched on the helix (aa 685–693) in the small N‐lobe. The tilt angles were determined between the planes along the strand (aa 872–875) and the helix (aa 825–836) in the C‐lobe. B) Western blot analysis of RAC1 ubiquitination by full length (FL) WT HACE1 and its E782A mutant using anti‐RAC1 antibody. C) Pair‐wise structural alignment of HUWE1 HECT domain (pink, aa 3991–4374, PDB:5LP8‐B) in closed conformation and HUWE1 HECT domain (green, aa 3991–4374, PDB:6XZ1‐A) in open conformation. D) Western blot analysis of RAC1 ubiquitination by FL WT HACE1 and its Δ3 CT mutant (aa 1–906) using anti‐RAC1 antibody. E) Multiple sequence alignment of the C‐terminal regions in human HECT E3 ligases. The arrow indicates the aromatic residue at −4 position. F) Western blot analysis of RAC1 ubiquitination by FL WT HACE1 and its Y906A mutant using anti‐RAC1 antibody. G) Western blot analysis of in vitro autoubiquitination by WT HACE1 extended HECT domain (aa 483–909) and its Δ3 CT mutant (aa 483–906) using anti‐ubiquitin antibody. H) Western blot analysis of in vitro autoubiquitination by WT HACE1 extended HECT domain (aa 483–909) and its Y906A mutant using anti‐ubiquitin antibody. I) E3‐Ub linkage of WT HACE1 extended HECT domain and its Δ3 CT and Y906A mutants detected by nonreducing SDS–PAGE and J) reducing SDS–PAGE visualized by Coomassie staining. Asterisk indicates the band corresponding to a mono‐Ub linked HACE1 extended HECT domain (aa 483–909).

Superimposition of the HECT domains of HACE1 monomer and dimer reveals the movement of the HECT domain C‐lobe by 17.2° which in turn causes the catalytic cysteine (C876) to slant by 14.7° (**Figure** **4**A). This movement is likely coordinated with the ≈5 Å increase in the gap of the HECT domain small N‐lobe induced by the insertion of the N‐terminal helix (aa 1–21) from the other subunit. It suggests that the restriction of the C‐lobe movement of the HECT domain is involved in ubiquitination activity. To identify the role of structural element regulating the C‐lobe movement, we performed structural alignment of the HECT domains from HACE1 monomer, AREL1 and E6AP (Figure S6A, Supporting Information). Glu782 is highly conserved among the HECT domains and maintains the closed‐conformation of the C‐lobe (Figure S6B, Supporting Information).^[^ ^10^ ^]^ We performed an in vitro RAC1 ubiquitination assay with the full‐length HACE1 bearing an E782A mutation. Interestingly, the E782A mutant did not alter HACE1 enzymatic activity (Figure 4B), which suggests that HACE1 rearranges the orientation of the C‐lobe by more than one intramolecular interaction.

Crystal structures of the human HUWE1 HECT domain (plus a 42‐residue extension) show that the C‐terminal tail (aa 4370–4374) of the HECT domain interacts with the ARL domain‐4 in a closed conformation and switches the contact to the upper N‐lobe of HECT domain in an open conformation (Figure 4C).^[^ ^42^, ^43^ ^]^ To test whether the C‐terminal tail (aa 906–909) of HACE1 has functional significance, we performed an in vitro RAC1 ubiquitination assay with a C‐terminal last 3‐residue deletion mutant (FL HACE1∆3CT; aa 1–906) (Figure 4D). We show that the FL HACE1∆3CT mutant lost its poly‐ubiquitination activity, indicating that the C‐terminal tail indeed plays a key role. Based on the high conservation of the aromatic residues at the −4 position among HECT E3 ligases (Figure 4E), we further created a FL HACE1 Y906A mutant. We observed RAC1 polyubiquitination completely abolished by this Y906A mutation (Figure 4F). To confirm that C‐terminal mutations affected catalytic activity, not autoinhibition or RAC1 binding, we generated an extended HACE1 HECT domain construct harboring only the HECT domain plus 40 aa at N‐terminal portion (aa 483–909), and performed in vitro autoubiquitination assay with and without C‐terminal mutations. The WT HACE1 HECT domain (aa 483–909) construct retained autoubiquitination activity, while the HACE1 HECT domain ∆3CT and Y906A mutants lost autoubiquitination activity (Figure 4G,H). These results demonstrate that the C‐terminal tail of the HACE1 E3 ligase is indeed involved in catalytic activity. Further, we sought to identify the exact step in the ubiquitination cascade which is hindered by these mutations. HECT E3 ligases autoubiquitinate or ubiquitinate substrates with a two‐step mechanism; the first step involves the conjugation of ubiquitin onto the HECT E3 catalytic cysteine forming a thioester bond which is susceptible to reducing condition. The second ubiquitin ligation step involves the formation of an iso‐peptide bond between ubiquitin and the substrate lysine, which tolerates reducing condition. To test it, we performed in vitro thioester formation assay using WT HACE1 HECT domain and its Δ3CT and Tyr906Ala mutants. The reactions were stopped in the absence and presence of a reducing agent (DTT) followed by SDS–PAGE analysis. The bands corresponding to E3–Ub show the thioester linked ubiquitin that is visible in the absence of DTT (Figure 4I). The deletion of last 3 C‐terminus residues and Y906A mutation significantly decreased transthiolation reaction compared to WT HACE1 HECT domain (Figure 4I,J). This observation indicates that E2‐E3 transthiolation step is impaired in Δ3CT and Y906A mutants which ultimately resulted in complete loss of enzymatic activity of HACE1 E3 ligase.

## 3 Discussion

HACE1, a HECT‐type E3 ligase, interacts with numerous substrates to facilitate a wide range of cellular functions. Yet, how it participates in so many interactions is unclear. Our cryo‐EM analysis revealed that HACE1 exists as a mixture of monomers and homodimers. Although HACE1 eluted as a dimer in the size‐exclusion chromatography, the cryo‐EM vitrification process may have broken a significant portion of dimer into monomer. We demonstrated that the N‐terminal helix mediates dimerization, and specifically, our HACE1 dimer structure shows that N‐terminal helix of one monomer blocks the putative E2‐conjugating enzyme binding site on the HECT domain of the other. Consequently, we demonstrate that deletion of the N‐terminal helix (∆21) results in a shift in the homodimeric‐monomeric equilibrium toward the monomer form in vitro, concomitant with a significant increase in RAC1 ubiquitination. Additionally, this increased ubiquitination is also likely attributed to enhanced interactions between ∆21 HACE1 and RAC1. Indeed, through HDX–MS analysis, we show that the AKR domain in the WT HACE1 does not interact with RAC1, whereas the ∆21 HACE1 makes significant interactions with RAC1 via its AKR repeats and MID domain. Notably, whereas others have suggested that HACE1 cannot perform autoubiquitination in vivo, we measured weak autoubiquitination of WT HACE1 and strong autoubiquitination of ∆21 HACE1 in vitro.^[^ ^44^ ^]^ Collectively, these findings illustrate how dimerization functions to control indiscriminate or unsolicited HACE1 enzymatic activity. Besides, we have also shown that the C‐terminal last three residues in the HECT domain of HACE1 play critical role during E2‐E3 transthiolation reaction and thus are important for regulating its enzymatic activity.

As we noted for HACE1, Smurf1, another NEDD4 subfamily HECT E3 ligase, forms an autoinhibited homodimer with intermolecular interactions between the N‐terminal C2 domain of one monomer and the HECT domain of the other. Previous work shows that Smurf1 dimerization is disrupted by the deletion of its C2 domains, leading to enhanced ubiquitination of its substrate RhoA in vitro.^[^ ^45^ ^]^ In addition, the Cdh1 E3 ubiquitin ligase disrupts the Smurf1 homodimer and activates Smurf1 independently of its E3 ligase activity. In our in vitro system, the deletion of N‐terminal helix dissociated the HACE1 dimer into monomers which substantially enhanced HACE1 enzymatic activity. Nevertheless, the cellular factors involved in shifting this equilibrium are unclear. We suggest that similar to Smurf1, HACE1 activity is regulated by homodimerization‐mediated autoinhibition. Furthermore, our findings suggest that intermolecular autoinhibition mechanisms may apply to other HECT‐type E3 ubiquitin ligases.

Indeed, various NEDD4 subfamily members have been shown to be activated via substrate‐dependent activation.^[^ ^46^, ^47^ ^]^ Smurf2, a NEDD4 subfamily HECT‐type E3 ligase, exists in autoinhibitory state as a monomer, in which its N‐terminal C2 domain is bound to its HECT domain.^[^ ^48^ ^]^ This autoinhibitory state of Smurf2 is relieved by binding of its substrate Smad7 to the WW domain adjacent to the HECT domain.^[^ ^47^ ^]^ Likewise, WWP1 and WWP2 from the NEDD4 subfamily exhibit removal of autoinhibition as part of its activation.^[^ ^48^, ^49^ ^]^ WWP1 and WWP2 have WW2‐linker‐WW3 module surrounding the HECT domain upper N‐lobe to lock the exo‐site (Figure S6C, Supporting Information). As a result, WWP1 and WWP2 are autoinhibited by blocking ubiquitin transfer. While we did not observe the dissociation of the HACE1 dimer to monomer upon binding with RAC1 (Figure S5E, Supporting Information), we are yet to identify what factors might activate dimeric HACE1 for effective substrate ubiquitination. HACE1 is likely autoinhibited by obstructing recruitment of the E2 conjugating enzyme. Indeed, our HACE1 structure shows that the N‐terminal helix1‐helix2 linker inserts into the E2‐conjugating enzyme binding groove.

Sedimentation velocity analysis and nonreducing SDS–PAGE suggest that WT HACE1 not only forms dimer but also higher oligomers, although we did not observe such particles exceeding 300 kD by cryo‐EM or size‐exclusion chromatography. Acosta et. al. showed that HACE1 phosphorylation at S385 by group I PAKs in vivo results in greater homo‐oligomerization but less ubiquitination of RAC1.^[^ ^44^ ^]^ This S385 residue is adjacent to the insertional loop (aa 386–441) of the MID domain, which we were unable to model. Structural alignment of the MID domain of monomeric and dimeric HACE1 shows the possibility of some rearrangement in this region when in a dimer form (Figure S6D,E, Supporting Information). Additionally, the previous study showed that the phospho‐mimetic HACE1 (S385E) mutant (greater oligomerization) had a reduced capacity to facilitate ubiquitination of active RAC1, and surmised that this site was therefore more likely to be linked to modulation of HACE1 enzymatic activity rather than target protein association.^[^ ^44^ ^]^ We speculate that higher oligomer of HACE1 is energetically unfavorable in vitro but can be stabilized by posttranslational modification in vivo as a part of regulatory mechanism.

**Figure 5:**
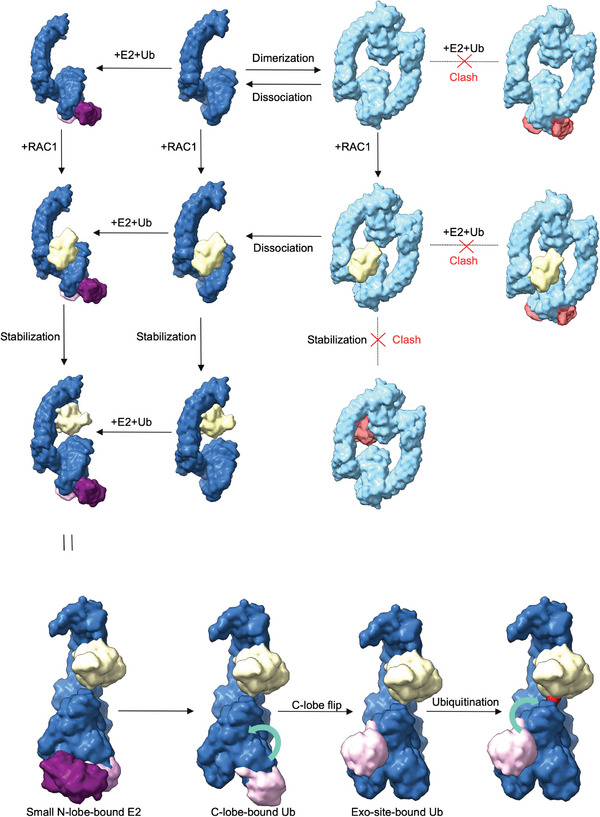
Schematic representation of autoinhibition, E2 binding, RAC1 binding and ubiquitination. HACE1 monomer (blue), HACE1 dimer (sky blue), ubiquitin conjugating enzyme E2 (purple), ubiquitin (pink), and RAC1 (yellow).

The structural and functional analyses of full‐length HACE1 underscore how oligomerization can regulate E3 ubiquitin ligase activity. As HACE1 form homodimers, its enzymatic activity is impaired, and its interaction with RAC1 or other cellular proteins may cause conformational changes that allow it to restore E3 ligase activity. Our MD simulation showed that HACE1 dimer is generally rigid and none of the docking models showed any possibility of RAC1 fitting in the dimeric ring of WT HACE1. The RAC1 structural dimension exceeds the gap between AKR (N‐term) and the HECT domain (C‐term) of the other subunit of the dimer, which could possibly explain why WT HACE1 does not interact strongly with RAC1. Furthermore, our HDX–MS analysis showed increased deuterium exchange in the AKR domain of WT HACE1 but decreased deuterium exchange in the AKR domain of ∆21 HACE1 upon binding RAC1 (Figure 3A,B). It implies that the AKR domain of ∆21 HACE1 was readily accessible to solvent, prior to binding RAC1. Furthermore, HDX–MS analysis, site directed mutagenesis, and in silico structure modeling suggest that RAC1 interact with WT HACE1 MID region, and the AKR might act as a scaffold to position RAC1 (**Figure** **5**). The breakdown of homodimers into monomers substantially enhances HACE1 interaction with RAC1 and its enzymatic activity. Besides, the C‐terminal last three residues of HACE1 play critical role during E2‐E3 transthiolation reaction and thus for its enzymatic activity. Due to the fact that HACE1 is misregulated in many cancers, our findings could pave the way for the development of therapeutic drugs that could modulate HACE1 dimerization to restore its tumor suppressor function.

## 4 Experimental Section

### Cloning, Protein Expression, and Purification

The protein constructs used in this study are listed in Supporting Information (Table S7, Supporting Information). Site‐directed mutagenesis PCR was performed to generate several HACE1 mutant constructs for in vitro ubiquitination assays. The clones were confirmed through DNA sequencing and subsequently expressed in *Escherichia coli BL21(DE3*).

Following protein expression, cells were harvested by centrifugation and lysed by sonication in lysis buffer (50 × 10^−3^ m Tris pH 8.0, 300 × 10^−3^ m NaCl, 5% glycerol) for all constructs. The lysate was cleared by centrifugation (18 000 rpm, 30 min, 4 °C, Beckman coulter JA20) and incubated with glutathione beads for 4 h at 4 °C. GST‐tagged full length HACE1 was purified by glutathione affinity chromatography after which GST tag was cleaved overnight with PreScission protease at 4 °C, followed by gel filtration chromatography with HEPES buffer (20 × 10^−3^ m HEPES pH 8.0, 150 × 10^−3^ m NaCl, 5% Glycerol, 1 × 10^−3^ m DTT). GST‐tagged ubiquitin, E1 (UBA1/UBE1), and E2 (UbcH7) were purified similarly by glutathione‐affinity chromatography and treated with PreScission Protease for overnight on‐column cleavage, followed by gel filtration chromatography. Similarly, the GST‐tagged RAC1 was purified, with the exception of ion‐exchange chromatography and gel filtration chromatography. Gel filtration and ion‐exchange chromatography procedures were conducted with the Superdex column (S200 and S75) and HiTrap Q HP anion exchange column respectively, using the ÄKTA pure chromatography system (GE Healthcare). The peak fractions were concentrated using Amicon centrifugal filter units. Protein concentrations were quantified via nanodrop, and homogeneity of protein samples was evaluated via dynamic light scattering experiments.

### Electron Microscopy Sample Preparation and Data Collection

Four microliters of the sample were applied on glow discharged UltrAufoil R1.2/1.3, and excess liquid was manually removed with filter paper. The process was repeated once more. The clamped grid was set in Vitrobot chamber, and 4 µL of the sample was applied the third time. After wait time 15 s, the grid was blotted for 3 s in 22 °C 100% humidity with force −5 and drain time 0 s. The grid was plunge‐frozen in liquid ethane cooled by liquid nitrogen. The frozen‐hydrated grid was loaded into Titan Krios cryo‐electron microscope equipped with Gatan K3 direct‐electron detector and operated at 300 keV. A 50‐frame movies were collected at 110 000× magnification in counting mode with a physical pixel size of 0.8584 Å pix^−1^. The images were recorded at defocus range of 0.5 to 2.5 µm, using SerialEM program (FEI; Thermo Fisher Scientific).

### Image Processing

The particles were extracted in Relion‐3.1.3 and selected in CryoSPARC‐3.2.0 by 2D classification, ab initio reconstruction and heterogeneous refinement.^[^ ^50^, ^51^ ^]^ The map and particles were exported to Relion‐3.1.3, Ctf‐refined and Bayesian polished.^[^ ^52^ ^]^ The polished particles were again selected in CryoSPARC‐3.2.0 by heterogeneous refinement. The selected particles were 3D autorefined, CtfRefined, and Bayesian polished three times. For HACE1 monomer, the polished particles were used for the final 3D autorefinement in Relion‐3.1.3 and postprocessed. For HACE1 dimer, the last polished particles were reconstructed again in CryoSPARC‐3.2.0 by Ab inito Reconstruction and Non‐Uniform Refinement. The particles were C2 symmetry‐expanded and refined by Local Refinement. The maps were sharpened in Phenix‐1.13 and modeled in ChimeraX‐1.0 and CCP‐EM‐1.6.0.^[^ ^53^, ^54^, ^55^, ^56^ ^]^ The initial models were derived from Alphafold and Robetta.^[^ ^57^, ^58^ ^]^ The models were rigid‐body fit in Chimera, rebuilt by Flex‐EM and ISOLDE and finally refined by Phenix.^[^ ^59^, ^60^, ^61^ ^]^ Refer to Supporting Information for the detailed workflow of single‐particle reconstruction (Figures S7 and S8, Supporting Information).

### In Vitro Ubiquitination Assays

Multiple in vitro ubiquitination assays were conducted to characterize the functional activity of full length HACE1 and its mutants. The reaction mixtures contain 0.05 × 10^−6^ m UBA1/UBE1, 1 × 10^−6^ m UbcH7, 3 × 10^−6^ m WT HACE1 or its mutants, 10 × 10^−6^ m Ubiquitin and 10 × 10^−6^ m constitutively active RAC1 (Q61L) as substrates. The ubiquitination mixtures were incubated at 37 °C for 60 min in 50 × 10^−3^ m HEPES pH 7.5, 100 × 10^−3^ m NaCl, 5 × 10^−3^ m ATP, 10 × 10^−3^ m MgCl_2_, 0.05 × 10^−3^ m DTT. The ubiquitination reaction was terminated with the addition of SDS–PAGE loading buffer prior to 12.5% SDS–PAGE analysis. For thioester formation analysis, HACE1 HECT (aa 483–909) and its C terminus mutants were incubated with E1, E2, Ub, and ATP for 2 min at 37 °C. The reactions were stopped in the absence and presence of a reducing agent (DTT) followed by SDS–PAGE analysis. The bands corresponding to E3–Ub show the thioester linked ubiquitin due to their susceptibility to reduction by DTT.

### Western Blot

SDS–PAGE gels were transferred to PVDF membranes (Sigma‐Aldrich) using a Tran‐ Blot SD Semi Dry Transfer Cell (Bio‐Rad). The blocking of membranes was performed with 5% BSA in phosphate buffered saline (PBS) containing 0.05% Tween 20 (PBS‐T) for 60 min. Membranes were then incubated with mouse monoclonal anti‐RAC1 or anti‐ubiquitin antibody (Santa Cruz Biotechnology, SantaCruz, CA) at 1:2000 dilutions at 4 °C overnight. Subsequently, membranes were subjected to three washes with PBS‐T and incubated with horseradish peroxidase (HRP) conjugated secondary antibodies (Jackson ImmunoResearch) in a 1:10 000 dilutions for 60 min at room temperature (23 °C). Membranes were further washed three times with PBS‐T and added with Super Signal West Pico Chemiluminescent Substrate (Thermo Scientific). Subsequent visualization was conducted with GeneSys image acquisition software with Syngene Pxi system.

### Mass Spectrometry

The ubiquitination reaction mixtures were resolved on 12.5% SDS–PAGE gel and stained with Coomassie Blue stain. The bands corresponding to ubiquitinated species were excised from the gels and subjected to Triple‐TOF 5600 MS analysis.

### Hydrogen‐Deuterium Exchange Mass Spectrometry (HDX–MS)

The interaction interface between HACE1 and RAC1 were determined using amide HDX–MS.^[^ ^62^ ^]^ For HDX labeling was carried out by diluting ≈75 pmol of purified proteins WT HACE1, ∆21 HACE1, and RAC1 individually in HEPES buffer prepared in deuterium oxide (final concentration D_2_O ≈90%). For HACE1–RAC1, ∆21 HACE1–RAC1 complexes, the two proteins were mixed in 1:3 stoichiometric ratio, incubated at 37 °C for 15 min prior to HDX labeling. Each HDX labeling was carried out for 1, 10, and 100 min timepoints. The reactions were stopped by lowering the pH_read_ ≈2.6 and 0 °C temperature, using a chilled quench solution (1% trifluoroacetic acid, 1.2 m guanidinium chloride, 0.2 m TCEP). For nondeuterated controls, individual proteins were diluted in aqueous HEPES buffer, immediately followed by quench and digestion.

Each quenched sample was subjected to online proteolysis using immobilized pepsin cartridge (Enzymate, Waters, USA) for 7 min at 12 °C temperature. The digested peptides were trapped onto a guard column (Vanguard C18 column) and then subjected to reverse‐phase liquid chromatography at near 0 °C, using nano‐ACQUITY M‐class (Waters) binary solvent manager, as described previously.^[^ ^63^, ^64^, ^65^ ^]^ The resolved and eluted peptides were then identified by a high‐resolution Synapt G2‐Si mass spectrometer (Waters, UK), operated in positive polarity mode. Peptides were ionized by electrospray ionization, spectra were collected by ion‐mobility HDMSe separation to ensure highest resolution, and mass accuracy maintained by continuous flow of Glu‐Fibrinogen peptide (100 fmol µL^−1^), as described previously.^[^ ^65^ ^]^

The peptides identified by MS were then matched and assigned using Protein Lynx Global Server 3.0 (Waters) using amino acid sequences of the proteins in individual databases. Peptides were loaded onto DynamX v3.0 software (Waters) and filtered using the following parameters—maximum sequence length: 25, intensity: 2000, and MH+ error ±10 ppm. Only peptides with high signal‐to‐noise ratio and nonoverlapping spectra were analyzed further. For each peptide, the deuterium uptake was measured at all labeling time points and was calculated as the differences between the mass centroid values of labeled and nondeuterated controls. The analyzed peptides were then manually checked for correct assignments and isotope distribution. The deuterium exchange values are represented as relative fractional uptake, which is the normalized ratio of the deuterium uptake by the peptide to the maximum exchangeable amide hydrogens in the peptide. HDX analysis yielded 194 peptides covering 93.4% sequence of ∆21 HACE1, 160 peptides covering 91% of full‐length HACE1, and 62 peptides encompassing 97.4% of RAC1. All measurements were done in triplicates, and the average deuterium uptake values are tabulated in the supplementary files (Table S8, Supporting Information). Statistical analysis and data visualization were done using Deuteros v2.0 software.^[^ ^66^ ^]^

### Sedimentation Velocity Analysis

Sedimentation velocity experiments were performed with a Beckman ProteomeLab XL‐1 analytical ultracentrifuge (Beckman Coulter, 253 Brea, California, USA). Epon double‐sector centerpieces equipped with quartz windows were filled with 400 µL and 380 µL of buffer and sample, respectively. After cell loading, alignment and temperature equilibration, the samples were centrifuged using an An50‐Ti rotor at 40 000 rpm and 20 °C until full sedimentation. Absorbance was scanned at 7 min interval. Analysis of the data was performed using UltraScanIII.^[^ ^67^ ^]^ The size distribution profile was fit in Continuous c(s) Distribution model.

### Molecular Dynamics Simulation

CABS‐flex 2.0 Server was used for coarse‐grained molecular dynamics simulations. Conformational sampling was conducted in Monte Carlo replica‐exchange dynamics, involving local movement of individual amino acids and global movement of small fragments. The simulation was run with default setting; 50 Monte Carlo cycles with 50 steps and temperature 1.4. GROMACS‐2023.1 package was used for all‐atom molecular dynamics simulations. The OPLS force field was applied to generate the essential topology records. The atomic coordinates were converted from the PDB format to the GROMACS format by pdb2gmx command with ‐ignh option to remove hydrogen atoms. The treated system was put in a cubical box and filled with SPC/E water molecules to solvate. Sodium ion was added to facilitate neutrality. The system was set to an equilibrium state using NPT and NVT ensembles with 50 000 steps. The all‐atom molecular dynamics simulation was carried out at 100 ns timescale by 0.002 ps X 50 000 000 steps. The obtained trajectories were applied to generate the PDB models.

## Conflict of Interest

The authors declare no conflict of interest.

## Supporting information

Supporting Information

Supporting Information

## Data Availability

The data that support the findings of this study are openly available in PDB at https://rcsb.org, EMDB at https://www.ebi.ac.uk/emdb/, reference number 8H8X, 8HAE, EMD‐34551 and EMD‐34586.
