# Supplementary material for: Structural Basis for the Enzymatic Activity of the HACE1 HECT‐Type E3 Ligase Through N‐Terminal Helix Dimerization
Source: Adv Sci (Weinh). 2023 Aug 3;10(27):2207672. doi: 10.1002/advs.202207672 (PMC10520629; doi:10.1002/advs.202207672)
Supplement: Supplementary file 1 — Supporting Information [file ADVS-10-2207672-s001.pdf]

ADVANCED SCIENCE
Open Access

Supporting Information

for *Adv. Sci.*, DOI 10.1002/advs.202207672

Structural Basis for the Enzymatic Activity of the HACE1 HECT-Type E3 Ligase Through N-Terminal Helix Dimerization

*Sunil Singh, Satoru Machida, Nikhil Kumar Tulsian, Yeu Khai Choong, Joel Ng, Srihari Shankar, Yaochen Liu, Krisha Vashdev Chandiramani, Jian Shi and J Sivaraman**

Supporting Information for

Structural basis for the enzymatic activity of the HACE1 HECT-type E3 ligase through N-terminal helix dimerization

*Sunil Singh[1], Satoru Machida[1], Nikhil Kumar Tulsian, Yeu Khai Choong, Joel Ng, Srihari Shankar, Liu Yaochen, Krisha Vashdev Chandiramani, Jian Shi, J Sivaraman**

[1]These authors contributed equally.

***Corresponding author**: J Sivaraman, **ORCID ID**: 0000-0001-9781-5326

Email: dbsjayar@nus.edu.sg

S. Singh, S. Machida, N. K. Tulsian, Y. K. Choong, J. Ng, S. Shankar, L. Yaochen, K. V. Chandiramani, J. Shi, J. Sivaraman

Department of Biological Sciences, National University of Singapore, 14 Science Drive 4, Singapore, 117543, Singapore

N. K. Tulsian,

Department of Biochemistry, National University of Singapore, 28 Medical Drive, Singapore, 117546, Singapore

This PDF file includes:

Figures S1 to S8
Tables S1 to S7

Other supporting materials for this manuscript include the following:

Table S8

Figure S1

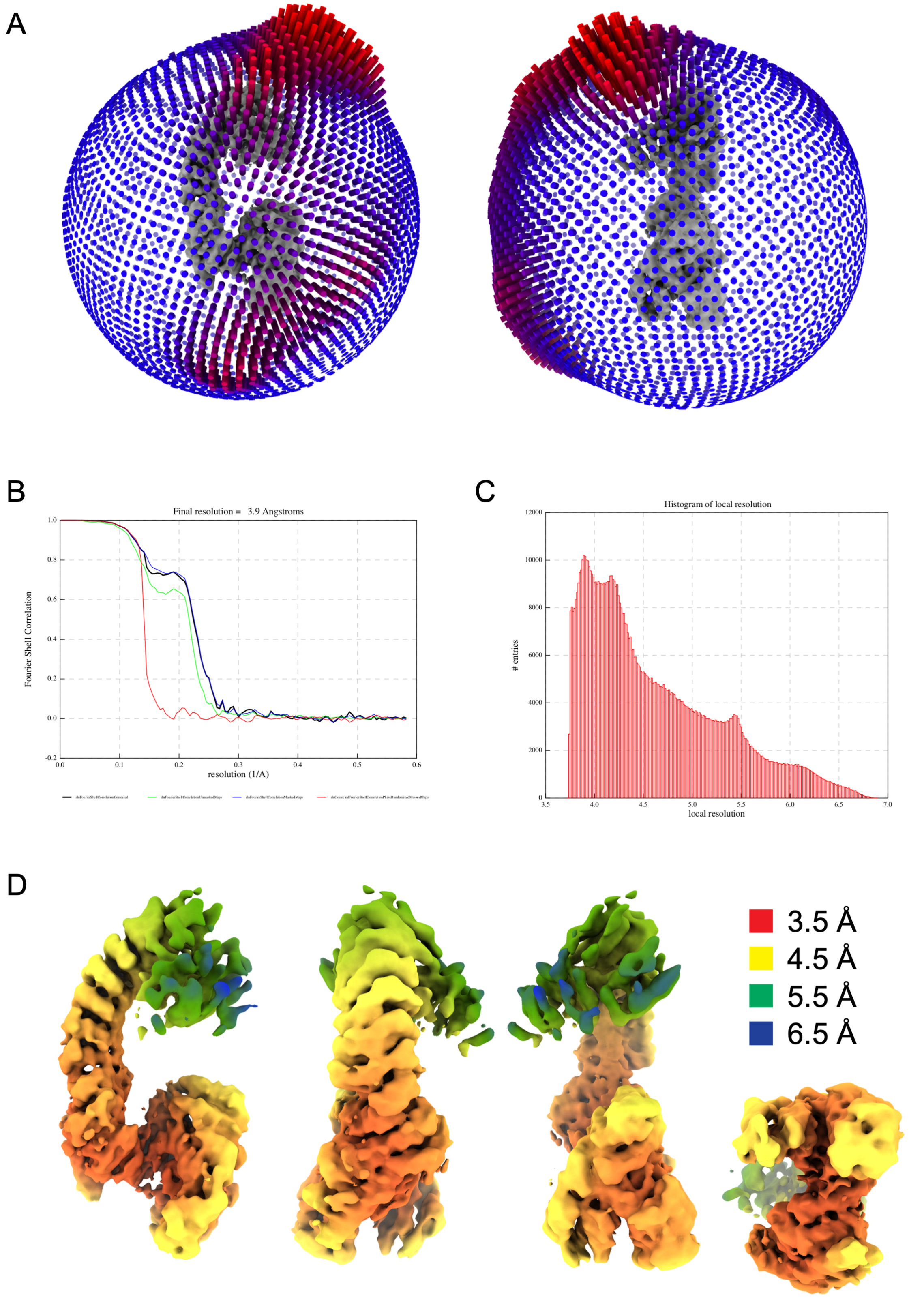

Figure S1. Local resolution estimation of HACE1 monomer. (A) Angular distribution of HACE1 monomer in the final refinement in Relion-3.1.3. **(B)** FSC plot by 0.143 cutoff. **(C)** Histogram of local resolution. **(D)** Coulomb potential map surface-colored with local resolution.

Figure S2

A

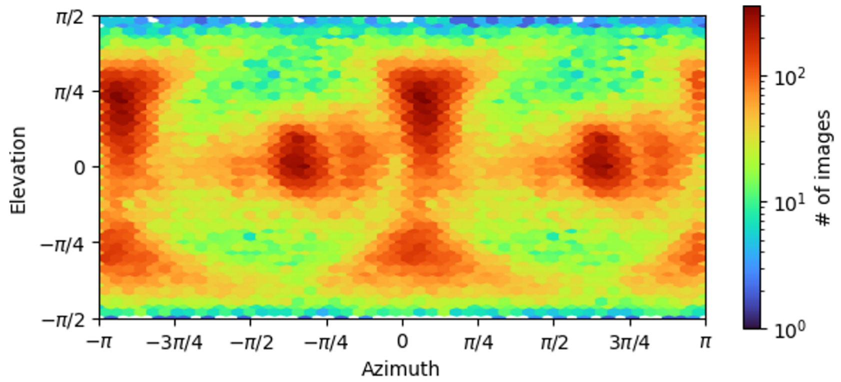

B

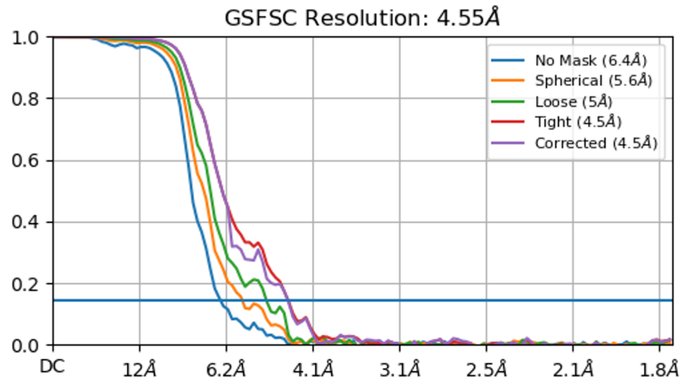

C

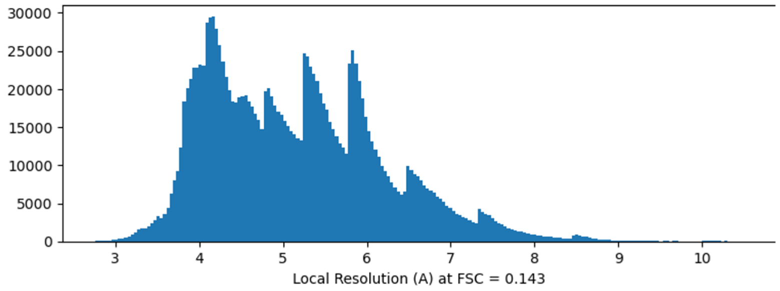

D

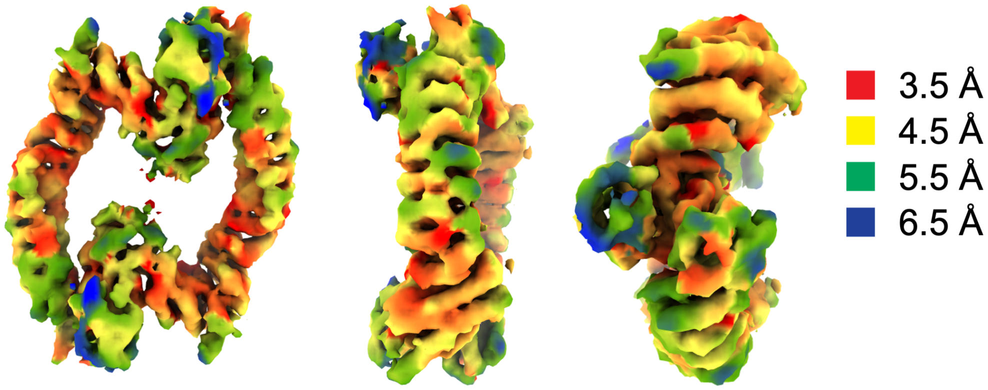

Figure S2. Local resolution estimation of HACE1 dimer. (A) Angular distribution of HACE1 dimer in the final refinement in CryoSPARC-3.2.0. **(B)** FSC plot by 0.143 cutoff. **(C)** Histogram of local resolution. **(D)** Coulomb potential map surface-colored with local resolution.

Figure S3

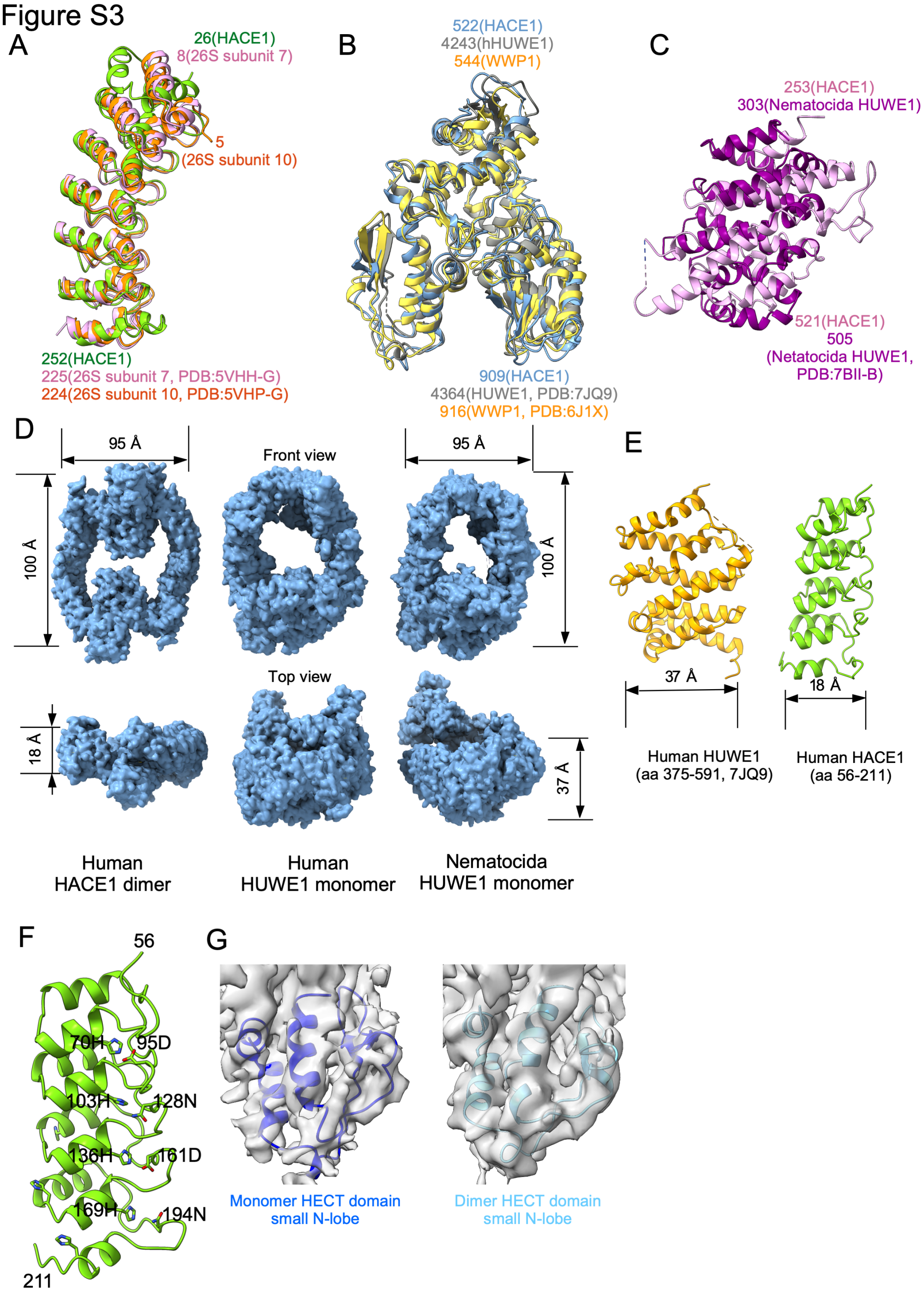

Figure S3. Structural homologues of HACE1 domains. (A) HACE1 AKR domain (light green, aa 26-252) superimposed on 26S proteasome regulatory subunit 10 (orange, PDB:5VHP-G, aa 5-224) and 26 proteasome regulatory subunit 7 (pink, PDB:5VHH-G, aa 8-225). **(B)** HACE1 HECT domain (light blue, aa 522-909) superimposed on human HUWE1 HECT domain (grey, PDB:7JQ9, aa 4243-4364) and WWP1 HECT domain (yellow, PDB:6J1X, aa 544-916). **(C)** HACE1 MID domain (pink, aa 253-521) superimposed on *Nematocida* HUWE1 (purple, PDB:7BII-B, aa 303-505) **(D)** Comparison of the dimensions of HACE1 dimer to HUWE1 monomer. **(Upper)** Front view. **(Lower)** Top view. **(E)** Comparison of the N-terminal solenoid of human HUWE1 (orange, aa 375-591) and HACE1 (light green, aa 56-211). **(F)** Regular positioning of histidine residues (aa 70, 103, 136, 169) facing connective loop containing Aspartate and Asparagine (aa 95, 128, 161, 194). **(G)** Comparison of the HECT domain N-lobes from HACE1 monomer and dimer. The width of the HECT domain small N-lobe in the model is consistent with the experimental map, to supplement Figure 2E.

Figure S4

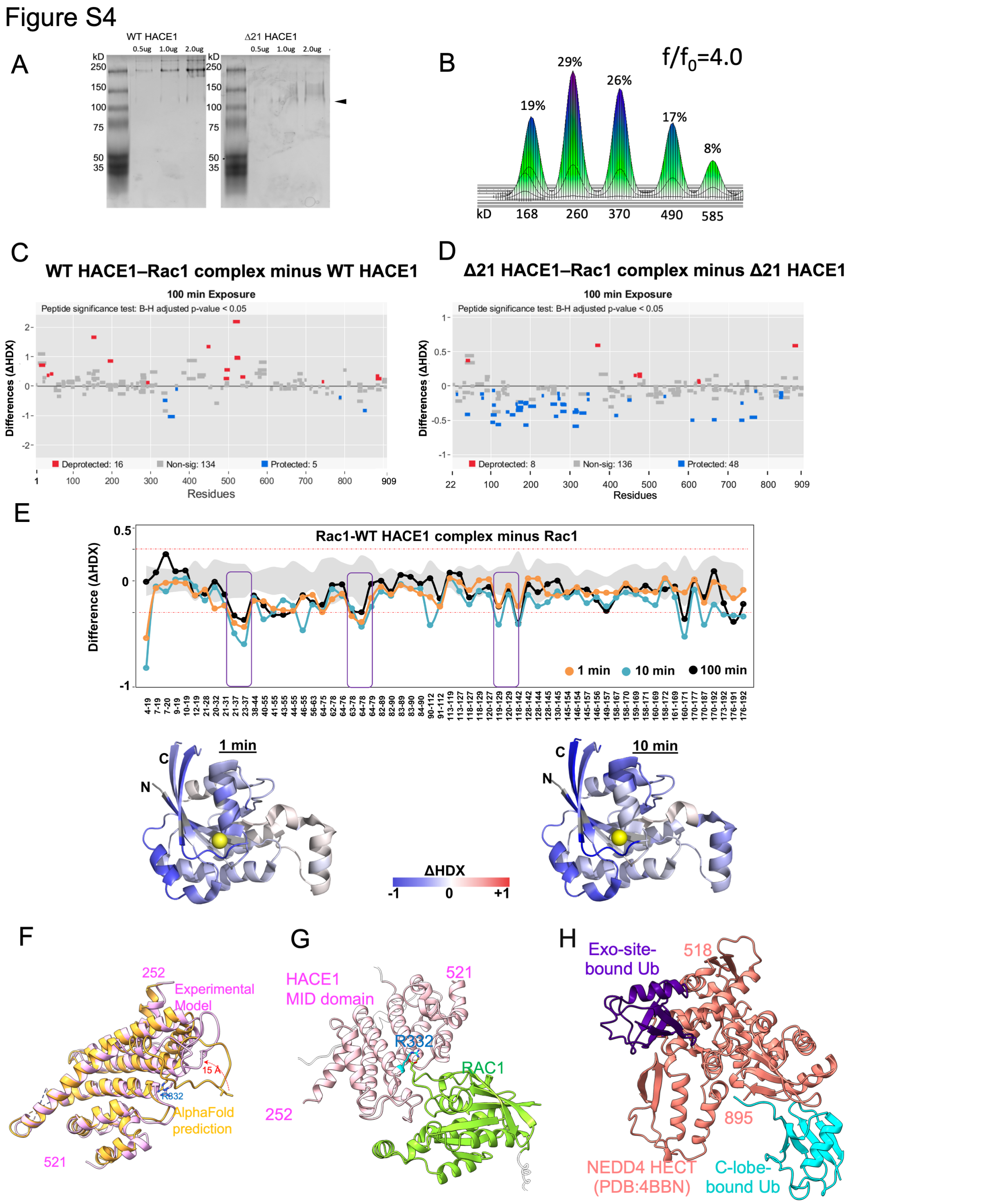

Figure S4. Mapping of HACE1-RAC1 interaction sites on HACE1 and RAC1. (A) Non-reducing SDS PAGE of WT and Δ21 HACE1. The triangle indicates the mobility of HACE1 monomer. **(B)** Sedimentation velocity analysis of WT HACE1. **(C)** Woods' differential plot showing deuterium exchange differences for WT HACE1+RAC1 relative to WT HACE1 alone at 100 min labeling time. **(D)** Woods' differential plot showing deuterium exchange differences for Δ21 HACE1+RAC1 relative to Δ21 HACE1 alone at 100 min labeling time. Pepsin-digested peptides are shown as horizontal bars, and were considered significant based on 99% confidence interval, analysed using Deuteros 2.0. **(E) (Upper)** Plots comparing the differences in deuterium exchange for RAC1 in the presence and absence of HACE1, at various labeling times indicated. Each dot represents a peptide of RAC1, with the residue numbers indicated. **(Lower)** Hydrogen-deuterium exchange differences mapped on the crystal structure of RAC1 (PDB: 4GZL), shown at 1 and 10 min timepoints. **(F)** Pair-wise structural alignment of WT HACE1 monomer MID domain (aa 252-521) and AlphaFold prediction (UniProt: Q8IYU2). **(G)** The interaction between WT HACE1 monomer and RAC1 at the MID domain, predicted by Galaxy Heteromer Server. The AKR and HECT domains are hidden for clarity. **(H)** Crystal structure of the NEDD4 HECT domain-Ubiquitin complex (PDB: 4BBN, aa 518-895).

Figure S5

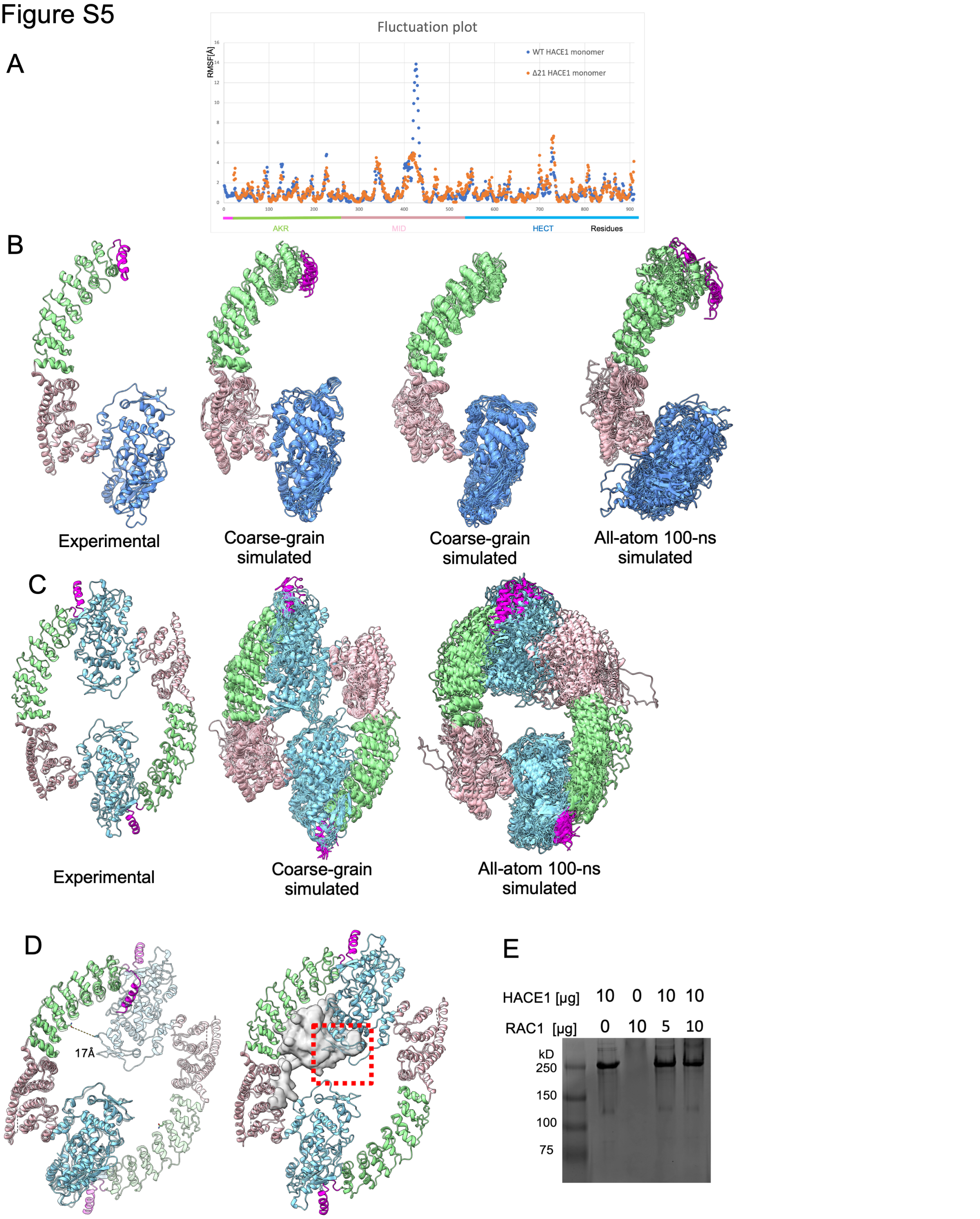

Figure S5. Molecular dynamics simulation of HACE1 monomer and dimer. (A) Fluctuation plot of WT and Δ21 HACE1 monomer from CABS-flex-2.0. **(B)** Trajectory cluster of HACE1 monomer models generated by CABS-flex 2.0 and Gromacs-2023.1. **(C)** Trajectory cluster of WT HACE1 dimer models generated by CABS-flex-2.0 and Gromacs-2023.1. **(D)** Potential clash of the HECT domain with RAC1 docked onto the AKR domain of WT HACE1 dimer. **(E)** Non-reducing SDS PAGE of WT HACE1 with or without RAC1.

Figure S6

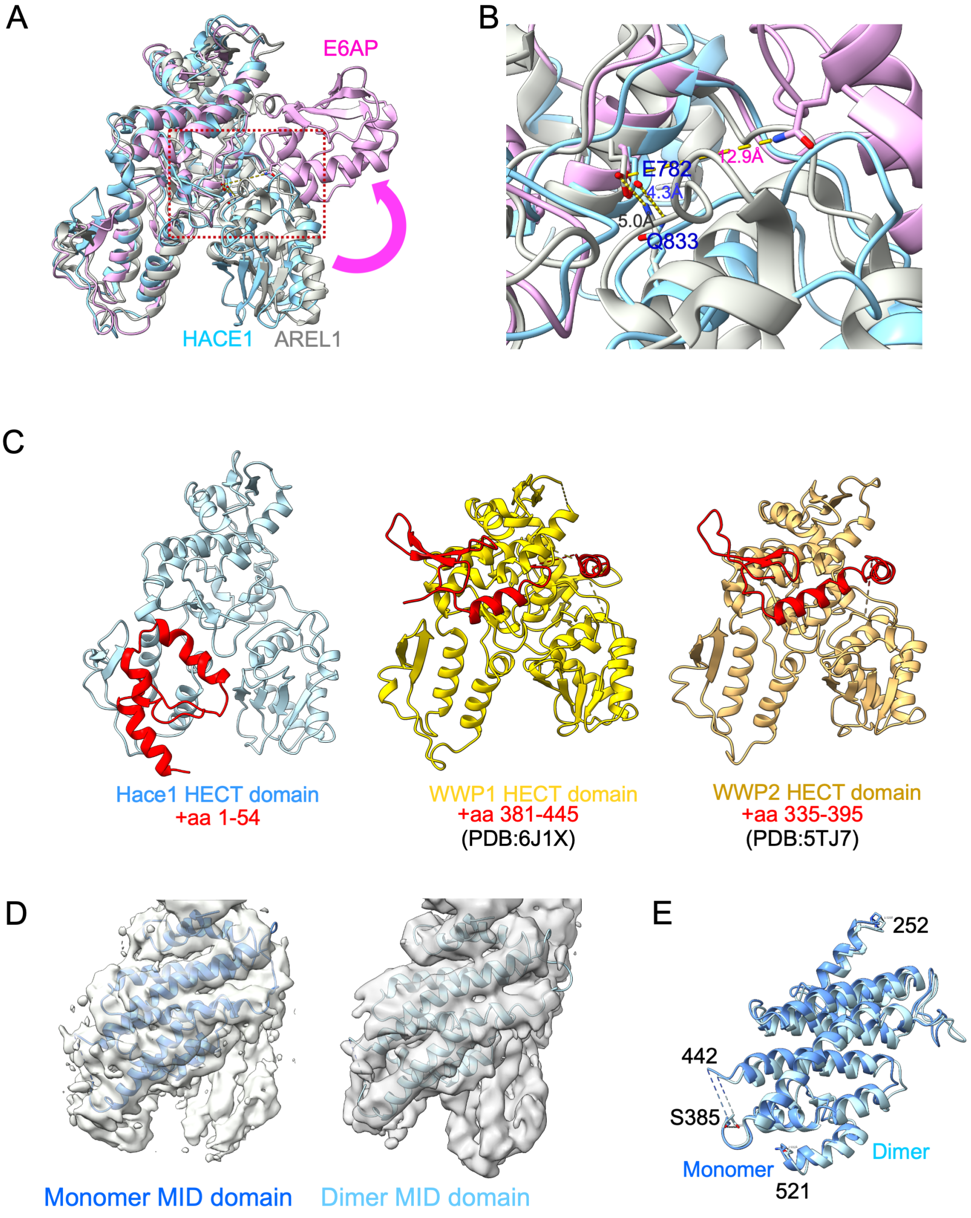

Figure S6. Domain rearrangement and auto-inhibition models. (A) Superimposed models of the HACE1 HECT domain (aa 522-909) and AREL1 HECT domains (PDB:6JX5, aa 437-814) in closed conformation and the E6AP HECT domain (PDB:1C4Z, aa 497-846) in open conformation. The boxed area is zoomed in on (B). **(B)** E782-Q833 interaction in the HACE1 HECT domain closed conformation and the equivalent interaction (E701-Q753) in AREL1. The E6AP HECT domain in open conformation has the equivalent residues E733 and Q784 too far apart to make the interaction. **(C)** Comparison of auto-inhibited HECT domains from HACE1, WWP1 and WWP2. **(Left)** HACE1 N-terminal helices and linkers (red) obstructs the HECT domain small N-lobe. **(Centre)** WWP1 WW2 domain plus linker (red) obstructs the HECT domain upper N-lobe. **(Right)** WWP2 WW2 domain plus linker (red) obstructs the HECT domain upper N-lobe. **(D)** Rearrangement of the HACE1 MID domain is coupled with dimerization. The insertion loop (aa 386-441) is hidden. **(E)** Structural alignment of the MID domains from the HACE1 monomer and dimer.

Figure S7

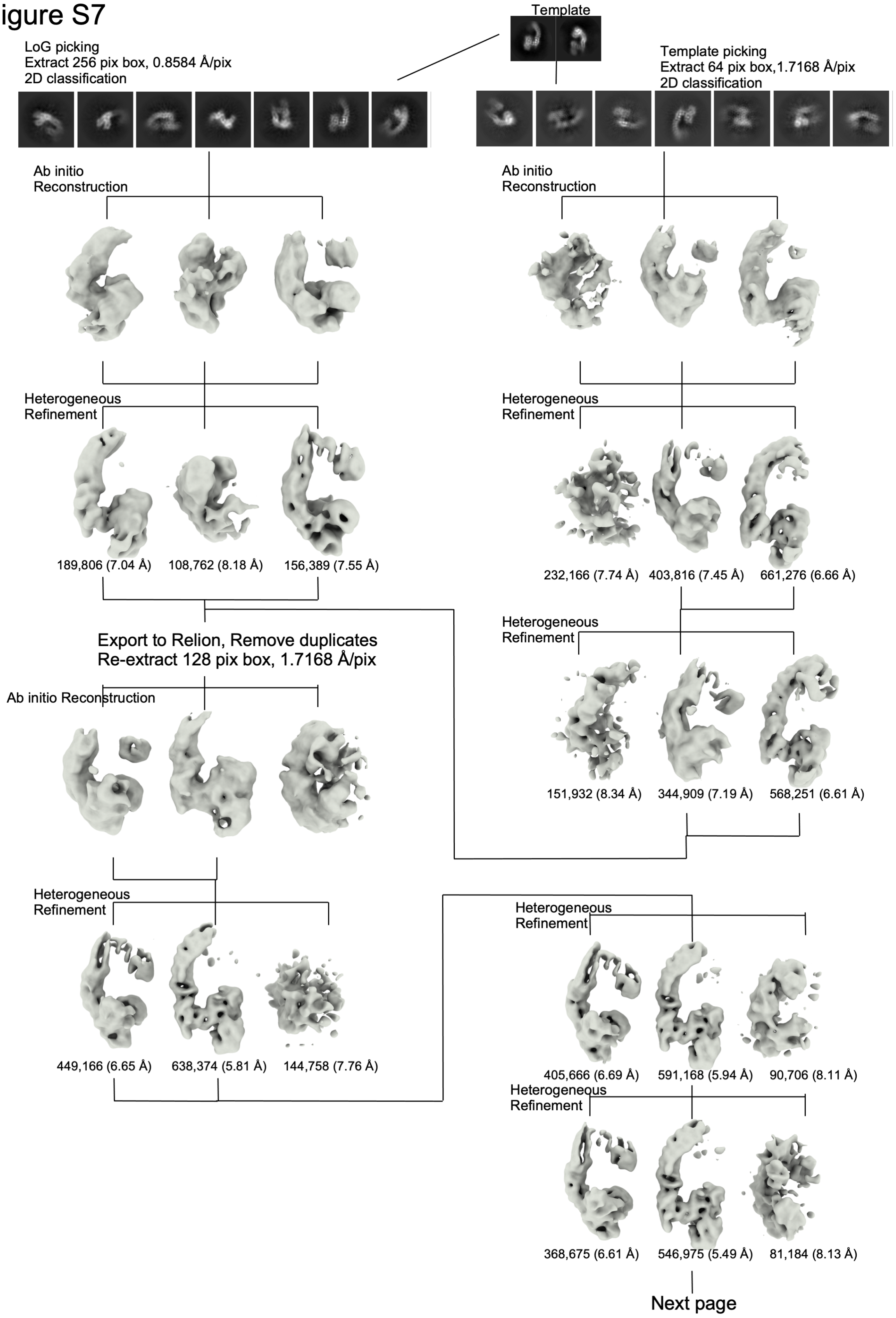
Template
LoG picking
Extract 256 pix box, 0.8584 Å/pix
2D classification
Template picking
Extract 64 pix box,1.7168 Å/pix
2D classification
Ab initio
Reconstruction
Ab initio
Reconstruction
Heterogeneous
Refinement
Heterogeneous
Refinement
189,806 (7.04 Å)
108,762 (8.18 Å)
156,389 (7.55 Å)
232,166 (7.74 Å)
403,816 (7.45 Å)
661,276 (6.66 Å)
Heterogeneous
Refinement
Export to Relion, Remove duplicates
Re-extract 128 pix box, 1.7168 Å/pix
Ab initio Reconstruction
151,932 (8.34 Å)
344,909 (7.19 Å)
568,251 (6.61 Å)
Heterogeneous
Refinement
Heterogeneous
Refinement
449,166 (6.65 Å)
638,374 (5.81 Å)
144,758 (7.76 Å)
405,666 (6.69 Å)
591,168 (5.94 Å)
90,706 (8.11 Å)
Heterogeneous
Refinement
368,675 (6.61 Å)
546,975 (5.49 Å)
81,184 (8.13 Å)
Next page

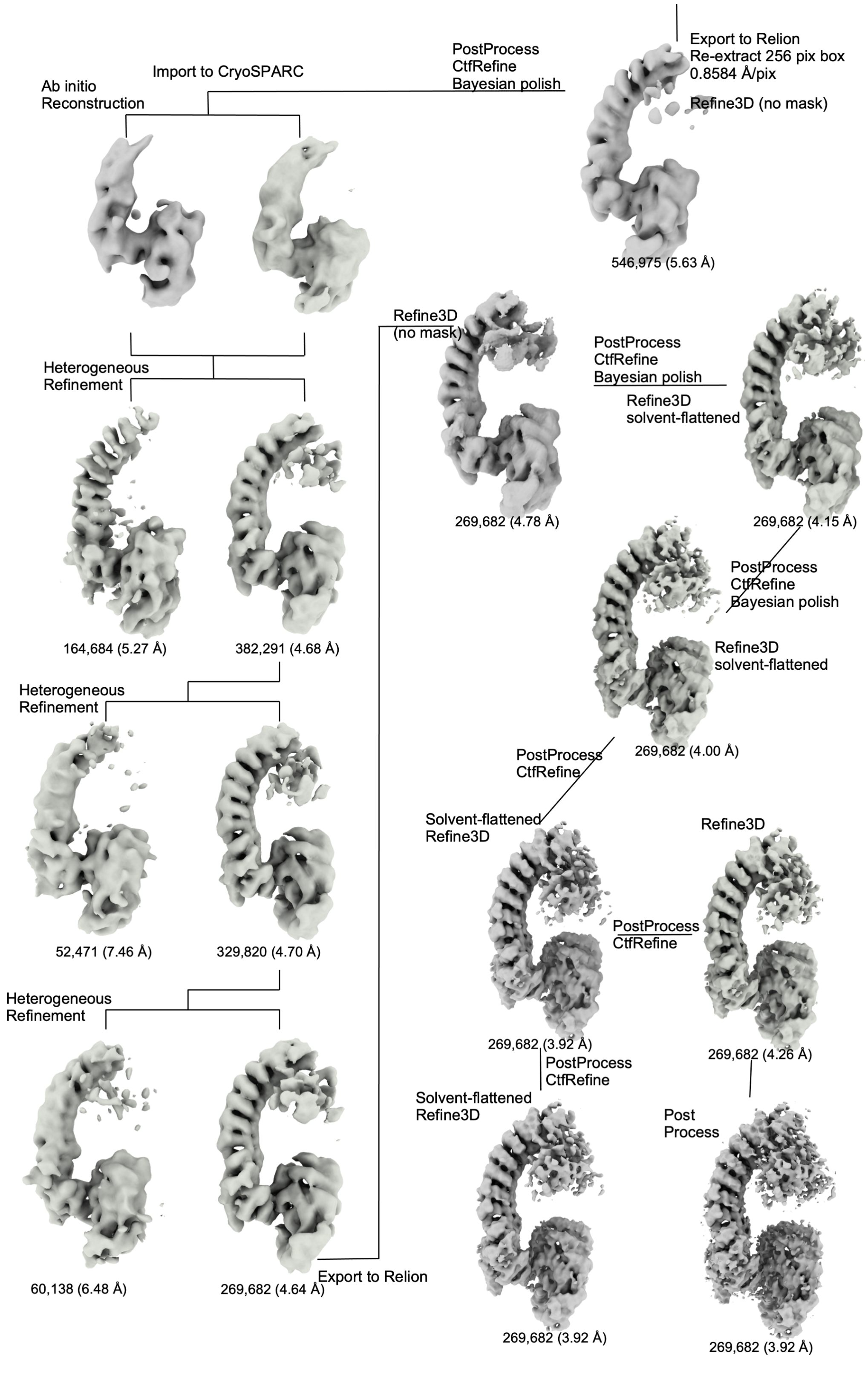

Note:
Class similarity 0 in all runs of Ab initio Reconstruction
Box rescaled to 128 pix in all runs of Heterogeneous Refinement

Figure S7. Image processing workflow for the HACE1 monomer.

The two datasets contained 10296 and 4984 stacks of movies without manual sorting. All images were imported to Relion-3.1.3, separately motion-corrected with dose-weighting by MotionCorr2 using Relion's own implementation and named opticsGroup1 and opticsGroup2. We first picked particles by Laplacian-of-Gaussian (LoG) and extracted 3,925,985 particles from opticsGroup2 with un-binned 0.8584 Å/pix, 256 pix^2/box, and imported to CryoSPARC-3.2.0. After 2 rounds of 2D classification, 454,957 particles were subjected to Ab-initio Reconstruction and Heterogeneous Refinement into 3 classes. Two 3D classes with combined 346,195 particles were retained for further processing. Meanwhile, 96,192 particles from selected 2D classes were exported to Relion-3.1.3 by pyem command csparc2star.py –copy-micrograph-coordinates and 2D classified again in Relion-3.1.3. The selected 2D averages were used as template to pick 8,500,730 particles from opticsGroup1 and -2. The templated-picked particles were extracted with fourfold binned 3.4336 Å/pix, rescaled from 256 to 64 pix^2/box and imported to CryoSPARC-3.2.0. Two rounds of 2D classification retained 1,297,258 particles for Ab-initio Reconstruction into 3 classes. 2 rounds of Heterogeneous Refinement selected two 3D classes with combined 913,160 particles. The 346,195 particles from LoG-picking and 913,160 particles from template-picking were exported to Relion-3.1.3 using pyem command csparc2star.py –copy-micrograph-coordinates and manually labelled with optics group name. The star files of separately exported particles were joined and selected, removing 27,057 duplicates by 30 Å distance cut-off. The remaining 1,232,298 particles were re-extracted with twofold binned 1.7168 Å/pix, rescaled from 256 to 128 pix^2/box, and imported to cryoSPARC-3.2.0. Ab-initio Reconstruction with 3 classes followed by 3 rounds of Heterogeneous Refinement retained 546,975 particles in one 3D class, which were exported to Relion-3.1.3 by pyem and re-extracted with un-binned 0.8584 Å/pix, 256 pix^2/box re-centred. The volume output of the best class from the last Heterogeneous Refinement was rescaled by Relion command relion_image_hander to 0.8584 Å/pix, 256 pix^2/box and imported to Relion-3.1.3 as 3D reference. The first 3D auto-refine without masking made a 5.63463 Å map, which was used to create a mask with low-pass 25 Å, binarization threshold 0.003, binary map extension 7 pix, and added soft-edge 8 pix. After post-processing, the particle from the first 3D auto-refine were CtfRefined successively fitting beam tilt, anisotropic magnification, and per-particle defocus and per-micrograph astigmatism. The subsequent Bayesian Polish was done separately for opticsGroup1 and -2 with trained parameters. The polished particles were imported to CryoSPARC-3.2.0 and divided into 2 classes by Ab-initio Reconstruction and Heterogeneous Refinement. The particles in the dominant 3D class were further reduced by 2 rounds of Heterogeneous Refinement. The remaining 269,682 particles were exported to Relion-3.1.3 by pyem without polishing information. The output volume from the Heterogeneous Refinement was rescaled and imported to Relion-3.1.3 and used as 3D reference for 3D auto-refine, making 4.77718 Å map without masking. After mask creation and post-processing, the particles were CtfRefined and Bayesian-polished in the aforementioned order. The second 3D auto-refine with mask made 4.14623 Å map using solvent-flattened FSC. The map was post-processed using the same mask and subjected to CtfRefine and Bayesian Polish in the same order as above. The 3D auto-refine after the second Bayesian Polish made 3.99546 Å map using solvent-flattened FSC. CtfRefine and 3D auto-refine were repeated twice more, resulting in 3.92411 Å map with or without solvent flattening followed by PostProcessing. Note that all the instances of Ab initio Reconstruction were run with class similarity=0. Heterogeneous Refinement automatically rescaled the box size to 128 pix^2.

Figure S8

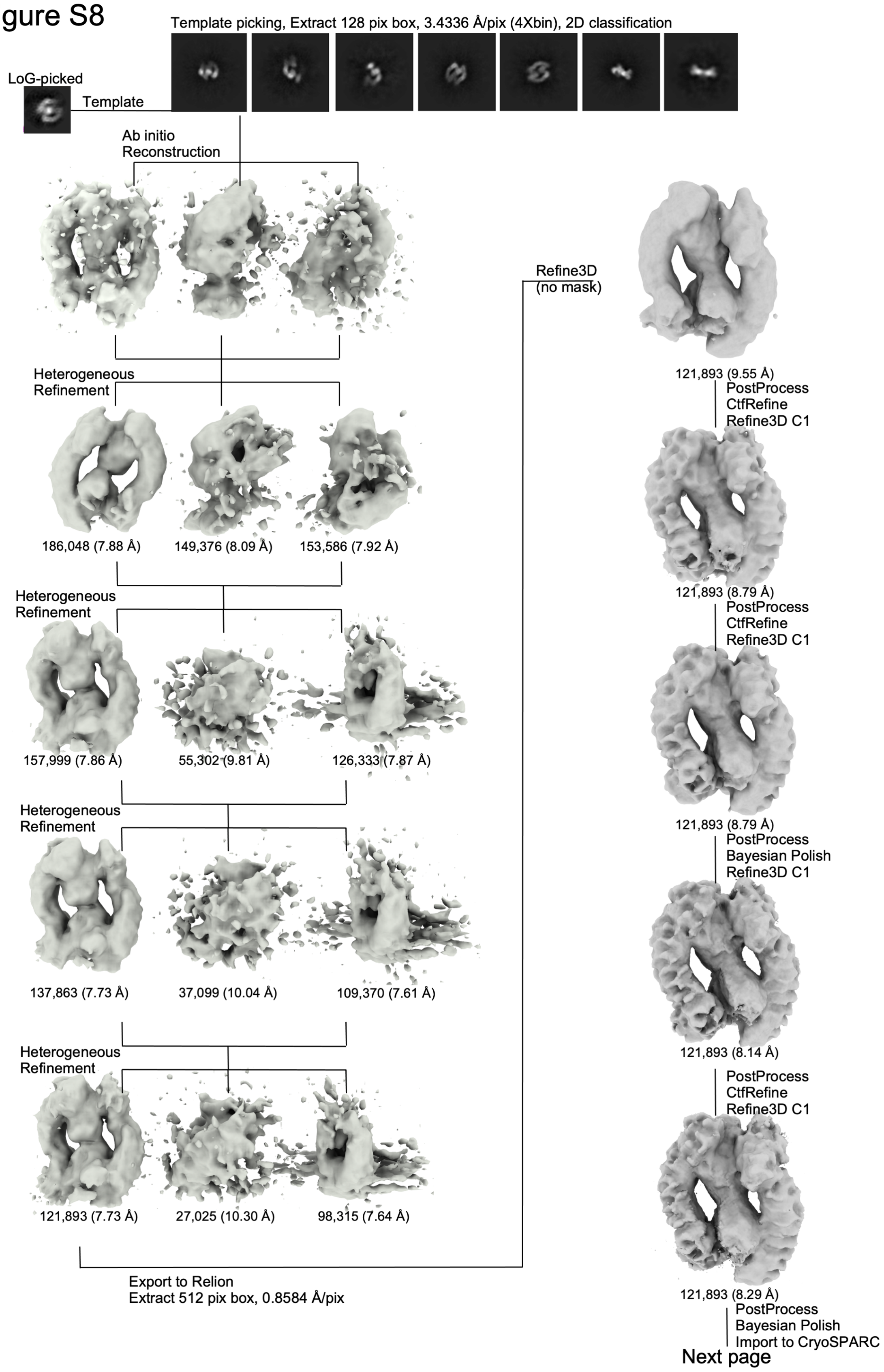

Template picking, Extract 128 pix box, 3.4336 Å/pix (4Xbin), 2D classification
LoG-picked
Template
Ab initio
Reconstruction
Heterogeneous
Refinement
186,048 (7.88 Å)
149,376 (8.09 Å)
153,586 (7.92 Å)
Heterogeneous
Refinement
157,999 (7.86 Å)
55,302 (9.81 Å)
126,333 (7.87 Å)
Heterogeneous
Refinement
137,863 (7.73 Å)
37,099 (10.04 Å)
109,370 (7.61 Å)
Heterogeneous
Refinement
121,893 (7.73 Å)
27,025 (10.30 Å)
98,315 (7.64 Å)
Export to Relion
Extract 512 pix box, 0.8584 Å/pix
Refine3D
(no mask)
121,893 (9.55 Å)
PostProcess
CtfRefine
Refine3D C1
121,893 (8.79 Å)
PostProcess
CtfRefine
Refine3D C1
121,893 (8.79 Å)
PostProcess
Bayesian Polish
Refine3D C1
121,893 (8.14 Å)
PostProcess
CtfRefine
Refine3D C1
121,893 (8.29 Å)
PostProcess
Bayesian Polish
Import to CryoSPARC
Next page

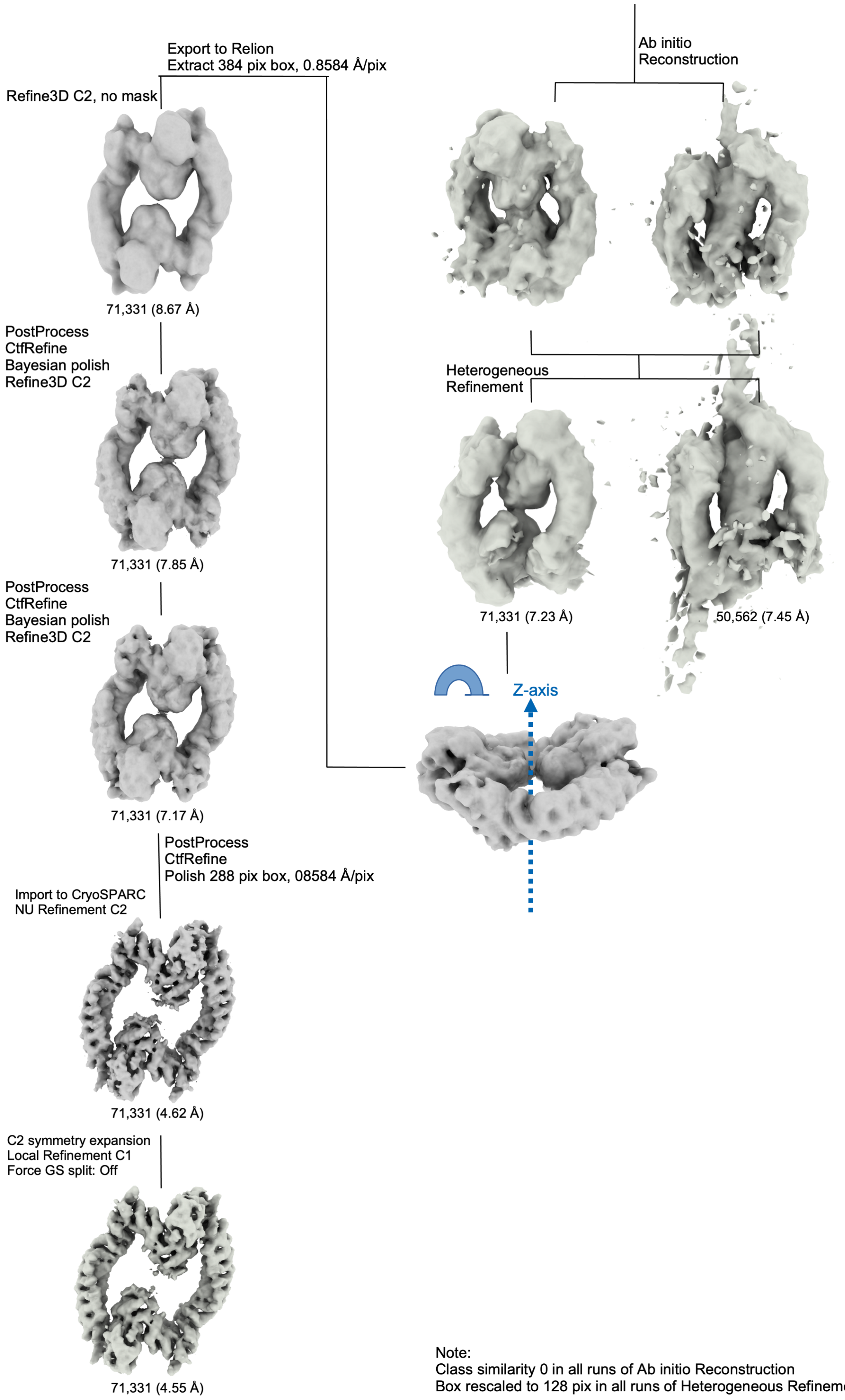

Note:
Class similarity 0 in all runs of Ab initio Reconstruction
Box rescaled to 128 pix in all runs of Heterogeneous Refinement

Figure S8. Image processing workflow for the HACE1 dimer.
LoG-picked 3,925,985 particles from opticsGroup2 with un-binned 0.8584 Å/pix, 256 pix^2/box, were imported to CryoSPARC-3.2.0 and 2D-classified. The 2D average containing HACE1 dimer were exported to Relion-3.1.3 by pyem command csparc2star.py –copy-micrograph-coordinates and 2D-classified again. The 2D average showing the complete shape of HACE1 dimer was used for template-picking. 4,000,808 particles were extracted with fourfold binned 3.4336 Å/pix, rescaled from 512 to 128 pix^2/box, and were imported to CryoSPARC-3.2.0. 489,010 particles were selected by two rounds of 2D-classification and used for Ab initio Reconstruction with 3 3D-classes. Four rounds of Heterogeneous Refinement identified one class containing 121,893 particles. They were exported to Relion-3.1.3 by pyem and re-extracted with un-binned 0.8584 Å/pix, 512 pix^2/box. The map from the last Heterogeneous Refinement was rescaled to 0.8584 Å/pix, 512 pix^2/box and used as 3D reference for 3D auto-refinement without mask, post-processed and CtfRefined fitting beam tilt, anisotropic magnification and per-particle defocus and per-micrograph astigmatism in this order, followed by another 3D auto-refinement. Post-processing, CtfRefine and 3D-autorefinement were repeated, followed by Bayesian polish and 3D-auto-refinement. Post-processing, CtfRefine, 3D-autorefine, post-processing and Bayesian polish were repeated. The particles were imported to CryoSPARC-3.2.0 and sorted by Ab initio Reconstruction and Heterogeneous Refinement with 2 3D-claasses. The particles in the dominant class were exported back to Relion-3.1.3 and re-extracted with un-binned 0.8584 Å/pix, 384 pix^2/box. The volume output from the last Heterogeneous Refinement was rescaled to 0.8584 Å/pix, 384 pix^2/box. The rescaled map was re-oriented, and the coordinates were resampled using ChimeraX command vop resample so that Z-axis pass through the center of the plane on which HACE1 dimer lay. The resampled map was used as the reference for 3D-auto-refinement with C2 symmetry imposed and no mask. Post-processing, CtfRefine, Bayesian polish, C2-imposed 3D auto-refine were repeated twice each time using a mask made from the last run of 3D auto-refinement. The box extraction size was changed to 288 pix^2 at the final Bayesian polish after Post-processing and CtfRefine. The map and particles were imported to CryoSPARC-3.2.0 and refined by Non-Uniform Refinement with auto-sharpening and C2 symmetry imposed, resulting in 4.62 Å map. The particles from C2-imposed Non-Uniform refinement was C2-expanded and subjected to Local Refinement with C1 using the whole mask and volume from Non-Uniform Refinement. The final resolution in Local Refinement was 4.55 Å and was used for modelling. The C1-refined map using C2-expanded particles was more interpretable than the C2-refined map, although the resolution did not greatly improve.

Table S1

Rank	PDB	Z	rmsd(Å)	lali	nres	%id	Description
1	5vhp-G	21.2	3.1	218	224	28	26S PROTEASOME NON-ATPASE REGULATORY SUBUNIT 10
2	5vhn-G	20.8	3	215	219	27	26S PROTEASOME NON-ATPASE REGULATORY SUBUNIT 10
3	5vhh-G	20.7	3	215	219	27	26S PROTEASOME REGULATORY SUBUNIT 7
4	5vhf-G	20.6	3	216	219	28	26S PROTEASOME NON-ATPASE REGULATORY SUBUNIT 10
5	1k1a-A	20.6	2.4	217	228	26	B-CELL LYMPHOMA 3-ENCODED PROTEIN
6	5vhq-G	20.5	3.1	216	220	28	26S PROTEASOME NON-ATPASE REGULATORY SUBUNIT 10
7	1k1b-A	20.5	2.3	218	228	26	B-CELL LYMPHOMA 3-ENCODED PROTEIN
8	1qym-A	20.3	3.1	217	223	28	26S PROTEASOME NON-ATPASE REGULATORY SUBUNIT 10
9	4nik-A	20.2	3.2	217	224	28	26S PROTEASOME NON-ATPASE REGULATORY SUBUNIT 10
10	7vo6-A	20.2	2.9	213	224	28	26S PROTEASOME NON-ATPASE REGULATORY SUBUNIT 10
11	5g4x-A	20.1	2.4	226	346	24	SH3 AND MULTIPLE ANKYRIN REPEAT DOMAINS
12	3b95-A	20.1	2.8	217	236	26	EUCHROMATIC HISTONE-LYSINE N-METHYLTRANSFERASE 1
13	5vhj-G	20.1	3.1	205	206	28	26S PROTEASOME NON-ATPASE REGULATORY SUBUNIT 10; PROTEIN 3
14	5w7j-C	20.1	2.9	219	231	25	PALMITOYLTRANSFERASE ZDHHC17
15	2dwz-A	20	3.2	217	226	28	26S PROTEASOME NON-ATPASE REGULATORY SUBUNIT 10
16	3aji-C	20	3.1	217	229	28	26S PROTEASOME NON-ATPASE REGULATORY SUBUNIT 10
17	2dwz-C	19.9	3.1	216	226	28	26S PROTEASOME NON-ATPASE REGULATORY SUBUNIT 10
18	3b7b-A	19.9	2.8	219	236	26	EUCHROMATIC HISTONE-LYSINE N-METHYLTRANSFERASE 1
19	6by9-A	19.8	2.9	222	268	27	HISTONE-LYSINE N-METHYLTRANSFERASE EHMT1
20	3aji-A	19.8	3.1	216	229	29	26S PROTEASOME NON-ATPASE REGULATORY SUBUNIT 10
21	5vhm-G	19.7	3.1	206	210	28	26S PROTEASOME NON-ATPASE REGULATORY SUBUNIT 10
22	5vhr-G	19.7	2.7	195	199	29	26S PROTEASOME NON-ATPASE REGULATORY SUBUNIT 10
23	3nbn-B	19.7	2.1	193	196	27	RECOMBINING BINDING PROTEIN SUPPRESSOR OF HAIRLES
24	6kyh-D	19.6	2.5	226	349	25	SH3 AND MULTIPLE ANKYRIN REPEAT DOMAINS
25	6kyh-A	19.6	2.4	225	349	24	SH3 AND MULTIPLE ANKYRIN REPEAT DOMAINS
26	6kyh-C	19.6	2.4	225	349	24	SH3 AND MULTIPLE ANKYRIN REPEAT DOMAINS PROTEIN 3
27	2dvw-A	19.6	3	217	229	29	26S PROTEASOME NON-ATPASE REGULATORY SUBUNIT 10; PROTEIN 3
28	6kyh-B	19.5	2.4	225	349	24	SH3 AND MULTIPLE ANKYRIN REPEAT DOMAINS PROTEIN 3
29	2dzn-A	19.5	3.5	213	226	27	PROBABLE 26S PROTEASOME REGULATORY SUBUNIT P28
30	5vkq-C	19.5	3	223	1499	20	NO MECHANORECEPTOR POTENTIAL C ISOFORM L; PROTEIN 3
31	6kyk-B	19.3	2.5	226	357	25	SH3 AND MULTIPLE ANKYRIN REPEAT DOMAINS
32	3b7b-B	19.2	3.2	214	224	27	EUCHROMATIC HISTONE-LYSINE N-METHYLTRANSFERASE 1
33	1oy3-D	19.1	2.1	195	220	28	TRANSCRIPTION FACTOR P65; PROTEIN 3
34	5vho-G	19	3	192	196	29	26S PROTEASOME NON-ATPASE REGULATORY SUBUNIT 10
35	1k3z-D	19	2.1	194	221	29	TRANSCRIPTION FACTOR P65
36	6ne4-B	19	2.6	190	194	35	DESIGNED REPEAT BINDING PROTEIN
37	5w7j-A	19	2.9	215	229	25	PALMITOYLTRANSFERASE ZDHHC17
38	2dzo-A	18.9	3.7	216	228	26	PROBABLE 26S PROTEASOME REGULATORY SUBUNIT P28
39	3b95-B	18.8	3.2	211	225	27	EUCHROMATIC HISTONE-LYSINE N-METHYLTRANSFERASE 1
40	3hra-A	18.8	2.7	190	199	29	ANKYRIN REPEAT FAMILY PROTEIN
41	5w7i-A	18.8	2.9	215	229	24	PALMITOYLTRANSFERASE ZDHHC17
42	6ne2-B	18.8	2.7	190	193	35	DESIGNED REPEAT PROTEIN BINDER
43	4o60-B	18.6	1.6	120	228	34	ANK-N5C-317
44	5vhi-G	18.6	2.5	188	226	25	26S PROTEASOME REGULATORY SUBUNIT 7
45	3eu9-C	18.6	2.8	210	226	26	HUNTINGTIN-INTERACTING PROTEIN 14
46	6ndz-D	18.4	2.7	185	190	37	FRIZZLED-8
47	5cbo-E	18.3	1.7	120	176	32	MBP3-16,IMMUNOGLOBULIN G-BINDING PROTEIN A
48	5cbo-K	18.2	1.7	120	176	32	MBP3-16,IMMUNOGLOBULIN G-BINDING PROTEIN A

Z : Normailized alignment score
rmsd : Root mean square deviation of the aligned portions
lali : The number of aligned residues
nres : The total number of residues in the model
%id : The percentage of sequence identify

Table S1. DALI search with the HACE1 AKR domain.

Table S2

Rank	PDB	Z	rmsd(Å)	lali	nres	%id	Description
1	5lp8-A	36.9	2.4	374	432	41	E3 UBIQUITIN-PROTEIN LIGASE HUWE1
2	3g1n-A	36.4	2.4	371	374	40	E3 UBIQUITIN-PROTEIN LIGASE HUWE1
3	3g1n-B	36	2.3	367	371	41	E3 UBIQUITIN-PROTEIN LIGASE HUWE1
4	5lp8-B	35.9	2.4	368	416	40	E3 UBIQUITIN-PROTEIN LIGASE HUWE1
5	4be8-A	35.9	2.6	366	378	40	E3 UBIQUITIN-PROTEIN LIGASE NEDD4 (A889F)
6	7jq9-A	35.8	2.3	366	2427	40	E3 UBIQUITIN-PROTEIN LIGASE HUWE1
7	5hpl-A	35.1	3.2	367	370	40	RSP5
8	5hpl-B	35	3.2	368	370	40	RSP5
9	5hps-A	34.8	2.6	366	374	39	WWP1 HECT
10	7mwf-A	34.8	2.3	366	2427	41	E3 UBIQUITIN-PROTEIN LIGASE HUWE1
11	1nd7-A	34.8	2.6	368	374	39	WW DOMAIN-CONTAINING PROTEIN 1
12	5c7m-A	33.9	2.7	360	370	41	E3 UBIQUITIN-PROTEIN LIGASE ITCHY HOMOLOG
13	5hpt-A	33.9	3.3	368	373	39	NEDD4-LIKE E3 UBIQUITIN-PROTEIN LIGASE WWP1
14	5tjq-A	33.9	2.6	359	361	38	NEDD4-LIKE E3 UBIQUITIN-PROTEIN LIGASE WWP2,NEDD4
15	7mop-A	33.8	2.2	357	2445	41	E3 UBIQUITIN-PROTEIN LIGASE HUWE1
16	7mwd-A	33.7	2.3	366	2427	40	E3 UBIQUITIN-PROTEIN LIGASE HUWE1
17	5hpt-G	33.7	2.9	363	367	39	NEDD4-LIKE E3 UBIQUITIN-PROTEIN LIGASE WWP1
18	6j1y-A	33.5	2.8	366	401	40	NEDD4-LIKE E3 UBIQUITIN-PROTEIN LIGASE WWP1
19	5hpt-D	33.4	3.2	366	371	40	NEDD4-LIKE E3 UBIQUITIN-PROTEIN LIGASE WWP1
20	5tj8-A	33.4	2.7	361	408	39	NEDD4-LIKE E3 UBIQUITIN-PROTEIN LIGASE WWP2,NEDD4
21	7mwe-A	32.6	2.4	366	2427	40	E3 UBIQUITIN-PROTEIN LIGASE HUWE1
22	5tj7-A	32.5	2.7	372	436	38	NEDD4-LIKE E3 UBIQUITIN-PROTEIN LIGASE WWP2
23	3h1d-A	32.4	2.8	356	382	41	E3 UBIQUITIN-PROTEIN LIGASE HUWE1
24	5tj7-C	32.4	2.7	371	434	39	NEDD4-LIKE E3 UBIQUITIN-PROTEIN LIGASE WWP2
25	5tj7-B	32.1	3	371	445	37	NEDD4-LIKE E3 UBIQUITIN-PROTEIN LIGASE WWP2
26	7bii-B	31.9	2.4	369	2144	39	E3 UBIQUITIN-PROTEIN LIGASE HUWE1
27	5tj7-D	31.5	3	368	445	36	NEDD4-LIKE E3 UBIQUITIN-PROTEIN LIGASE WWP2
28	4y07-A	31.3	2.4	329	333	39	NEDD4-LIKE E3 UBIQUITIN-PROTEIN LIGASE WWP2
29	6j1z-A	31	2.3	334	403	39	NEDD4-LIKE E3 UBIQUITIN-PROTEIN LIGASE WWP2
30	6jx5-A	30.7	3.3	358	378	32	APOPTOSIS-RESISTANT E3 UBIQUITIN PROTEIN LIGASE 1
31	3tug-A	30.5	3	331	334	40	E3 UBIQUITIN-PROTEIN LIGASE ITCHY HOMOLOG
32	6jx5-B	30.5	3.3	359	378	31	APOPTOSIS-RESISTANT E3 UBIQUITIN PROTEIN LIGASE 1
33	6j1x-B	30.4	2.6	366	472	39	NEDD4-LIKE E3 UBIQUITIN-PROTEIN LIGASE WWP1
34	6jx5-C	30.4	3.5	358	378	32	APOPTOSIS-RESISTANT E3 UBIQUITIN PROTEIN LIGASE 1
35	6loh-A	29.6	3.5	354	373	33	APOPTOSIS-RESISTANT E3 UBIQUITIN PROTEIN LIGASE 1
36	6loh-B	29.5	3.8	355	375	32	APOPTOSIS-RESISTANT E3 UBIQUITIN PROTEIN LIGASE 1
37	6j1y-B	29.2	2.4	348	464	37	NEDD4-LIKE E3 UBIQUITIN-PROTEIN LIGASE WWP1
38	6loh-C	28.7	4	352	372	32	APOPTOSIS-RESISTANT E3 UBIQUITIN PROTEIN LIGASE 1
39	5xmc-A	28.3	2.6	309	409	38	E3 UBIQUITIN-PROTEIN LIGASE ITCHY
40	7nh3-A	27	2.4	366	2138	39	E3 UBIQUITIN-PROTEIN LIGASE HUWE1
41	7bii-A	26.6	2.4	367	2143	39	E3 UBIQUITIN-PROTEIN LIGASE HUWE1
42	5c91-A	21.7	9.3	298	375	38	E3 UBIQUITIN-PROTEIN LIGASE NEDD4
43	1zvd-A	21.6	12.6	319	373	37	SMAD UBIQUITINATION REGULATORY FACTOR 2
44	6xz1-A	21.5	14	313	384	41	HECT, UBA AND WWE DOMAIN CONTAINING 1, ISOFORM
45	6xz1-B	21.3	14	313	384	42	HECT, UBA AND WWE DOMAIN CONTAINING 1, ISOFORM
46	2oni-A	21.2	3.5	273	377	38	E3 UBIQUITIN-PROTEIN LIGASE NEDD4-LIKE PROTEIN
47	5hpk-A	21.2	3.9	277	372	37	E3 UBIQUITIN-PROTEIN LIGASE NEDD4-LIKE
48	2xbf-A	21.2	6.1	277	375	35	E3 UBIQUITIN-PROTEIN LIGASE NEDD4

Z : Normailized alignment score
rmsd : Root mean square deviation of the aligned portions
lali : The number of aligned residues
nres : The total number of residues in the model
%id : The percentage of sequence identify

Table S2. DALI search with the HACE1 HECT domain.

Table S3

Rank	PDB	Z	rmsd(Å)	lali	nres	%id	Description
1	5cmw-A	7.9	3.5	118	158	5	EPSIN-5
2	2x6k-A	7.8	10	95	550	8	PHOSPHOTIDYLINOSITOL 3 KINASE 59F
3	4n5a-A	7.5	3.8	128	535	11	PROTEIN EFR3
4	5cmy-A	7.5	3.5	114	142	6	EPSIN-5
5	2x6f-B	7.5	8.7	94	542	10	PHOSPHOTIDYLINOSITOL 3 KINASE 59F
6	5cmy-B	7.4	3.7	116	142	8	EPSIN-5
7	4ct4-A	7.4	3.5	150	236	7	CCR4-NOT TRANSCRIPTION COMPLEX SUBUNIT 1
8	2x6i-A	7.4	10.1	95	546	8	PHOSPHOTIDYLINOSITOL 3 KINASE 59F
9	6tps-O	7.4	3.5	162	463	12	DNA-DIRECTED RNA POLYMERASE I SUBUNIT RPA190
10	5anr-A	7.3	3.5	152	245	7	CCR4-NOT TRANSCRIPTION COMPLEX SUBUNIT 1
11	4crw-A	7.3	3.5	152	226	7	CCR4-NOT TRANSCRIPTION COMPLEX SUBUNIT 1
12	5n61-O	7.3	3.5	148	463	10	DNA-DIRECTED RNA POLYMERASE I SUBUNIT RPA190
13	1qgk-A	7.2	3.9	159	876	6	PROTEIN (IMPORTIN BETA SUBUNIT)
14	4ct4-C	7.2	3.6	150	242	7	CCR4-NOT TRANSCRIPTION COMPLEX SUBUNIT 1
15	6rrd-O	7.2	3.5	162	504	14	TEMPLATE STRAND
16	1he8-A	7.2	12.7	93	749	9	PHOSPHATIDYLINOSITOL 3-KINASE CATALYTIC SUBUNIT
17	4kzc-A	7.2	11	95	821	8	PHOSPHATIDYLINOSITOL 4,5-BISPHOSPHATE 3-KINASE CA
18	3ihy-A	7.2	10.2	94	536	5	PHOSPHATIDYLINOSITOL 3-KINASE CATALYTIC SUBUNIT
19	5g2n-A	7.2	11.8	100	823	10	PHOSPHATIDYLINOSITOL-4,5-BISPHOSPHATE 3-KINASE CA
20	7rsp-A	7.2	10.1	96	528	5	PHOSPHATIDYLINOSITOL 3-KINASE CATALYTIC SUBUNIT T
21	5jhb-A	7.2	10.9	94	811	11	PHOSPHATIDYLINOSITOL 4,5-BISPHOSPHATE 3-KINASE CA
22	5enn-A	7.2	13.1	102	526	6	PHOSPHATIDYLINOSITOL 3-KINASE CATALYTIC SUBUNIT T
23	6rui-O	7.2	3.5	162	504	14	TEMPLATE STRAND
24	2p8q-A	7.1	4	166	873	7	IMPORTIN BETA-1 SUBUNIT
25	7rsv-B	7.1	13.1	103	534	7	PHOSPHATIDYLINOSITOL 3-KINASE CATALYTIC SUBUNIT T
26	2a4z-A	7.1	10.9	95	812	11	PHOSPHATIDYLINOSITOL-4,5-BISPHOSPHATE 3-KINASE CA
27	7jwe-A	7.1	8.4	90	815	11	PHOSPHATIDYLINOSITOL 4,5-BISPHOSPHATE 3-KINASE CA
28	3g2w-B	7.1	2.9	114	138	6	ADP-RIBOSYLATION FACTOR-BINDING PROTEIN GGA1
29	3g2s-B	7.1	3.1	116	141	7	ADP-RIBOSYLATION FACTOR-BINDING PROTEIN GGA1
30	1jwf-A	7.1	3.1	115	139	7	ADP-RIBOSYLATION FACTOR BINDING PROTEIN GGA1
31	3ldz-C	7	3.3	117	140	8	UBIQUITIN
32	4gmj-A	7	3.5	150	229	8	CCR4-NOT TRANSCRIPTION COMPLEX SUBUNIT 1
33	4ajw-A	7	11.1	86	765	13	PHOSPHATIDYLINOSITOL-4,5-BISPHOSPHATE 3-KINASE CA
34	2a5u-A	7	11.8	100	839	12	PHOSPHATIDYLINOSITOL-4,5-BISPHOSPHATE 3-KINASE CA
35	5kae-A	7	8.6	94	840	9	PHOSPHATIDYLINOSITOL 4,5-BISPHOSPHATE 3-KINASE CA
36	3g2w-A	7	2.7	112	139	7	ADP-RIBOSYLATION FACTOR-BINDING PROTEIN GGA1
37	7wa4-A	7	3.1	112	529	7	PROTEIN GIGANTEA
38	7oba-O	6.9	3.6	148	347	10	DNA-DIRECTED RNA POLYMERASE I SUBUNIT RPA1
39	3ldz-A	6.9	3.3	116	140	8	UBIQUITIN
40	3ldz-D	6.9	3.4	116	140	9	UBIQUITIN
41	3ldz-B	6.9	3.4	117	140	9	UBIQUITIN
42	4gml-A	6.9	3.5	150	235	8	CCR4-NOT TRANSCRIPTION COMPLEX SUBUNIT 1
43	1py1-A	6.9	2.8	113	142	7	ADP-RIBOSYLATION FACTOR BINDING PROTEIN GGA1
44	2ntx-A	6.9	4.6	86	314	8	EMB|CAB41934.1
45	1py1-B	6.9	3	114	139	6	ADP-RIBOSYLATION FACTOR BINDING PROTEIN GGA1
46	2chx-A	6.9	11.3	102	842	8	PHOSPHATIDYLINOSITOL-4,5-BISPHOSPHATE 3-KINASE
47	5fi4-A	6.9	12.6	91	1009	10	PHOSPHATIDYLINOSITOL 4,5-BISPHOSPHATE 3-KINASE CA
48	6mum-B	6.9	12.8	93	822	12	PHOSPHATIDYLINOSITOL 4,5-BISPHOSPHATE 3-KINASE CA

Z : Normailized alignment score
rmsd : Root mean square deviation of the aligned portions
lali : The number of aligned residues
nres : The total number of residues in the model
%id : The percentage of sequence identify

Table S3. DALI search with the HACE1 MID domain.

Table S4

Solvation energy [kcal/mol]	Subunit-1		Subunit-2	
Isolated structure	-752.8	100.0%	-752.9	100.0%
Gain on complex formation	-7.7	1.0%	-8.1	1.1%
Average gain	-8.6	1.1%	-8.7	1.2%
P-value	0.579		0.557	
**Number of residues**	**Subunit-1**		**Subunit-2**	
Interface	78	9.2%	77	9.1%
Surface	840	99.4%	841	99.5%
Total[a]	845	100.0%	845	100.0%
**Surface area[Å]**	**Subunit-1**		**Subunit-2**	
All	47213.6.8	100.0%	47212.7	100.0%
Buried	2420.9	5.1%	2418.2	5.1%
Buried total	4839.1 (5.40%)			

[a]Residue count excludes MID loop

Table S4. HACE1 dimerization interface analysed by PDBePISA.

Table S5

Datalog Table: WT HACE1 (1.48 mg/ml) Reading 1

	Item	Time (s)	Intensity (Cnt/s)	DLS Temp (C)	Radius (nm)	Amplitude	%PD	Mw-R (kDa)	Baseline	SOS	Cuvette	Mw-S (Da)
1	Acq 1	2.6e+02	323267	25.0	7.4	0.437	14.8	363.4	1.000	11.799	JC-604	139031.07
2	Acq 2	2.7e+02	313473	25.0	7.2	0.433	17.7	338.0	1.000	11.001	JC-604	138030.83
3	Acq 3	2.8e+02	310621	25.0	7.5	0.436	14.7	374.0	1.000	15.985	JC-604	137313.82
4	Acq 4	2.8e+02	304470	25.0	7.3	0.434	17.7	347.8	1.000	8.446	JC-604	137427.19
5	Acq 5	2.9e+02	311403	25.0	7.4	0.432	16.4	360.3	1.001	15.678	JC-604	137528.79
6	Acq 6	2.9e+02	314989	25.0	7.4	0.431	12.2	365.4	1.000	9.388	JC-604	137721.32
7	Acq 7	3e+02	315247	25.0	7.5	0.433	16.7	375.6	1.000	11.808	JC-604	137388.60
8	Acq 8	3e+02	316725	25.0	7.4	0.437	19.5	361.1	1.000	11.248	JC-604	137249.52
9	Acq 9	3.1e+02	316095	25.0	7.5	0.429	15.9	374.0	1.000	10.673	JC-604	137773.76
10	Acq 10	3.1e+02	300665	25.0	7.5	0.436	12.8	378.3	1.000	14.621	JC-604	137596.70
Mean			312696	25.0	7.4	0.434	15.8	363.8	1.000	12.065		137706.16
S			6414	0.0	0.1	0.003	2.3	12.9	0.000	2.562		521.77
%S			2	0.0	1.5	0.629	14.3	3.5	0.025	21.238		0.38
S²			41143160	0.0	0.0	0.000	5.1	166.5	0.000	6.566		272240.50
Min			300665	25.0	7.2	0.429	12.2	338.0	1.000	8.446		137249.52
Max			323267	25.0	7.5	0.437	19.5	378.3	1.001	15.985		139031.07

Datalog Table: Δ21 HACE1 (1.48 mg/ml) Reading 1

	Item	Time (s)	Intensity (Cnt/s)	DLS Temp (C)	Radius (nm)	Amplitude	%PD	Mw-R (kDa)	Baseline	SOS	Cuvette	Mw-S (Da)
1	Acq 1	7.4e+04	197554	25.0	6.3	0.420	16.0	248.5	1.000	21.095	JC-604	90893.32
2	Acq 2	7.4e+04	191088	25.0	6.3	0.414	11.0	252.1	1.000	27.258	JC-604	90958.12
3	Acq 3	7.4e+04	202579	25.0	6.3	0.406	13.6	253.8	1.000	20.171	JC-604	90987.00
4	Acq 4	7.4e+04	198946	25.0	6.4	0.402	13.1	257.2	0.999	30.981	JC-604	91160.84
5	Acq 5	7.4e+04	202703	25.0	6.6	0.406	11.2	273.9	1.000	21.130	JC-604	91173.80
6	Acq 6	7.4e+04	206156	25.0	6.4	0.417	15.6	262.3	1.000	24.750	JC-604	91019.62
7	Acq 7	7.4e+04	196381	25.0	6.4	0.409	15.1	262.9	1.000	22.047	JC-604	90972.75
8	Acq 8	7.4e+04	202513	25.0	6.5	0.414	10.4	265.2	1.000	28.819	JC-604	90947.67
9	Acq 9	7.4e+04	197201	25.0	6.5	0.423	11.4	272.4	1.000	30.136	JC-604	90986.52
10	Acq 10	7.4e+04	203066	25.0	6.4	0.421	11.6	257.2	1.000	24.359	JC-604	90925.05
Mean			199819	25.0	6.4	0.413	12.9	260.6	1.000	25.075		91002.47
S			4406	0.0	0.1	0.007	2.1	8.4	0.000	4.007		93.68
%S			2	0.0	1.4	1.749	16.2	3.2	0.028	15.981		0.10
S²			19411169	0.0	0.0	0.000	4.4	70.2	0.000	16.058		8775.82
Min			191088	25.0	6.3	0.402	10.4	248.5	0.999	20.171		90893.32
Max			206156	25.0	6.6	0.423	16.0	273.9	1.000	30.981		91173.80

Table S5. Static Light Scattering analysis of WT HACE1 and △21 HACE1.

Table S6

Domain	aa	Count	Domain	aa	Count	Domain	aa	Count	Domain	aa	Count	Domain	aa	Count	Domain	aa	Count
AKR	M5I	1	AKR	L224M	1	MID	E369K	1	MID	M512V	1	HECT	L657V	1	HECT	E826K	1
AKR	V21M	1	AKR	D226H	2	MID	E377A	1	MID	M512I	1	HECT	R664Q	2	HECT	V835G	1
AKR	A30D	1	AKR	L233Q	1	MID	M379T	1	MID	F515C	1	HECT	S665F	1	HECT	T836M	1
AKR	P36L	1	AKR	G240R	3	MID	K380N	1	MID	H517L	1	HECT	G672C	1	HECT	S838N	2
AKR	A40T	1	AKR	G242V	1	MID	D384N	1	MID	Q522H	1	HECT	P674T	1	HECT	R840M	1
AKR	Q42K	1	AKR	V247L	1	MID	S385T	1	HECT	R527C	4	HECT	P674H	1	HECT	R840S	2
AKR	S45F	1	AKR	I249L	1	MID	T389A	1	HECT	R527H	1	HECT	D679N	1	HECT	G845V	1
AKR	F55Y	1	AKR	Y251H	1	MID	T389S	2	HECT	R527P	1	HECT	A681T	2	HECT	M850V	1
AKR	R63H	1	AKR	H252N	1	MID	G397D	2	HECT	C528R	2	HECT	N690D	1	HECT	M850I	1
AKR	S67I	1	MID	P253S	1	MID	Q398K	2	HECT	E533K	1	HECT	N690I	1	HECT	M850I	1
AKR	I71T	1	MID	P253Q	2	MID	D399G	1	HECT	E533A	1	HECT	N690K	1	HECT	G854V	1
AKR	A72T	1	MID	I259T	1	MID	A403D	1	HECT	P540A	1	HECT	L691W	1	HECT	A861T	3
AKR	G76V	1	MID	T263A	1	MID	A403V	1	HECT	D543N	1	HECT	S700R	1	HECT	P864S	1
AKR	S77T	1	MID	T263I	1	MID	P406S	1	HECT	R547S	1	HECT	G703S	1	HECT	P867S	1
AKR	S77L	2	MID	E266K	1	MID	P411Q	1	HECT	E551K	1	HECT	E711Q	1	HECT	L869R	1
AKR	E79V	1	MID	L268V	1	MID	G412V	1	HECT	H558Y	1	HECT	E719K	1	HECT	P871Q	1
AKR	D95G	1	MID	E270Q	1	MID	P413H	1	HECT	H558R	1	HECT	E719D	1	HECT	S873L	1
AKR	D95V	1	MID	M272I	2	MID	P413L	1	HECT	I562L	1	HECT	P722S	1	HECT	C876R	1
AKR	G98D	1	MID	R274Q	2	MID	G414E	1	HECT	R564T	1	HECT	P722R	1	HECT	N878D	2
AKR	T100K	1	MID	R274L	1	MID	S415I	1	HECT	S571T	1	HECT	G727S	1	HECT	Y885C	1
AKR	A105E	1	MID	Q275K	1	MID	Y416C	1	HECT	G581W	1	HECT	A738V	3	HECT	I890M	1
AKR	G109W	1	MID	S281C	1	MID	T421S	1	HECT	G581V	1	HECT	Y740H	1	HECT	G902D	1
AKR	Q110H	1	MID	Q287R	1	MID	G422D	1	HECT	A583V	1	HECT	V741L	1	HECT	G905D	1
AKR	M114V	1	MID	Q287H	1	MID	T423P	1	HECT	R585W	1	HECT	V741F	1	HECT	Y906C	1
AKR	A122P	1	MID	Y288H	1	MID	K427T	1	HECT	H587Y	1	HECT	R751T	1			
AKR	A122S	1	MID	K290N	1	MID	A437D	1	HECT	G591D	1	HECT	Q756R	1			
AKR	D123H	1	MID	A296V	1	MID	A439S	1	HECT	R598C	1	HECT	I757M	1			
AKR	N129H	1	MID	G303C	1	MID	A439G	1	HECT	R598H	1	HECT	A759V	1			
AKR	L132I	1	MID	H304D	1	MID	D443Y	1	HECT	D602N	1	HECT	L761F	1			
AKR	L132F	1	MID	H304R	1	MID	A449G	1	HECT	D602E	1	HECT	Q762H	1			
AKR	R143W	2	MID	S310F	1	MID	R451W	1	HECT	N610I	1	HECT	G763D	4			
AKR	R143Q	2	MID	S311I	1	MID	R451L	1	HECT	P611R	2	HECT	P769L	1			
AKR	T144A	1	MID	Q316E	1	MID	A458V	1	HECT	D612E	1	HECT	P770R	1			
AKR	D149N	1	MID	L320I	1	MID	M461T	1	HECT	Q618K	1	HECT	P770L	1			
AKR	V154D	3	MID	R325I	1	MID	C462Y	1	HECT	G622V	1	HECT	L775R	1			
AKR	E160V	1	MID	V330I	1	MID	M467I	2	HECT	T623R	1	HECT	Y779F	2			
AKR	E160D	1	MID	R332Q	5	MID	P469Q	1	HECT	T624P	1	HECT	V793L	1			
AKR	A162V	2	MID	S339I	1	MID	P469L	1	HECT	Q626R	2	HECT	S794N	1			
AKR	H169Y	1	MID	M344T	1	MID	R475H	1	HECT	S629N	1	HECT	S794T	1			
AKR	Q173L	2	MID	G345S	1	MID	E478K	1	HECT	N630D	2	HECT	D795G	2			
AKR	G175S	1	MID	K350T	1	MID	F479S	2	HECT	V633I	1	HECT	N799K	1			
AKR	L184P	1	MID	P352S	1	MID	C481S	1	HECT	L638F	1	HECT	Y806F	1			
AKR	G187D	1	MID	R353G	1	MID	D484Y	1	HECT	N639Y	1	HECT	W815L	3			
AKR	R192S	1	MID	R353K	1	MID	R493I	1	HECT	Y640H	1	HECT	F816L	2			
AKR	S196L	1	MID	Q355H	1	MID	N494H	1	HECT	R642W	4	HECT	W817R	1			
AKR	G197R	1	MID	P359S	2	MID	P495S	4	HECT	R642Q	1	HECT	D822Y	1			
AKR	S206T	1	MID	E361K	2	MID	K496Q	1	HECT	A651T	1	HECT	I823V	1			
AKR	A221T	1	MID	D368N	1	MID	E507D	1	HECT	A651V	1	HECT	T824I	1			
AKR	Y223S	1	MID	D368V	1	MID	L511V	1	HECT	R655W	1	HECT	Q825L	1			

Table S6. Mis-sense substitution mutations in HACE1 listed in COSMIC v96.

Table S7

Constructs	Description
FL HACE1 (aa 1-909)	Full length HACE1 was cloned into a pGEX6P-1 vector with GST tag
Δ21 HACE1 (aa 22-909)	HACE1 (aa 22-909) was cloned into a pGEX6P-1 vector with GST tag
FL HACE1 Y906A	FL HACE1 Tyr906Ala was cloned into a pGEX6P-1 vector with GST tag
FL HACE1 Δ3CT	FL HACE1 Δ3CT (aa 1-906) was cloned into a pGEX6P-1 vector with GST tag
FL HACE1 R332A	FL HACE1 Arg332Ala was cloned into a pGEX6P-1 vector with GST tag
FL HACE1 R353A	FL HACE1 Arg353Ala was cloned into a pGEX6P-1 vector with GST tag
FL HACE1 P359A	FL HACE1 Pro359Ala was cloned into a pGEX6P-1 vector with GST tag
FL HACE1 V140A	FL HACE1 Val140Ala was cloned into a pGEX6P-1 vector with GST tag
FL HACE1 Q173A	FL HACE1 Gln173Ala was cloned into a pGEX6P-1 vector with GST tag
FL HACE1 N174A	FL HACE1 Asn174Ala was cloned into a pGEX6P-1 vector with GST tag
FL HACE1 G175S	FL HACE1 Gly332Ser was cloned into a pGEX6P-1 vector with GST tag
FL HACE1 A204T	FL HACE1 Ala204Thr was cloned into a pGEX6P-1 vector with GST tag
Δ21 HACE1 R332A	Δ21 HACE1 Arg332Ala was cloned into a pGEX6P-1 vector with GST tag
Δ21 HACE1 R353A	Δ21 HACE1 Arg353Ala was cloned into a pGEX6P-1 vector with GST tag
Δ21 HACE1 P359A	Δ21 HACE1 Pro359Ala was cloned into a pGEX6P-1 vector with GST tag
Δ21 HACE1 V140A	Δ21 HACE1 Val140Ala was cloned into a pGEX6P-1 vector with GST tag
Δ21 HACE1 Q173A	Δ21 HACE1 Gln173Ala was cloned into a pGEX6P-1 vector with GST tag
Δ21 HACE1 N174A	Δ21 HACE1 Asn174Ala was cloned into a pGEX6P-1 vector with GST tag
Δ21 HACE1 G175S	Δ21 HACE1 Gly332Ser was cloned into a pGEX6P-1 vector with GST tag
Δ21 HACE1 A204T	Δ21 HACE1 Ala204Thr was cloned into a pGEX6P-1 vector with GST tag
RAC1 Q61L	Full length RAC1 Gln61Leu was cloned into a pGEX6P-1 vector with GST tag
E2	UbcH7 was cloned into a pGEX6P-1 vector with GST tag
E1	Human UBA1 was cloned into a pGEX6P-1 vector with GST tag
Ub	Ubiquitin was cloned into a pGEX6P-1 vector with GST tag

Table S7. List of constructs used in this study.

Separate Excel file

Table S8. Sequences, counts and deuterium uptake values of peptides detected in HDX-MS analysis.
